# Weighted Echo State Graph Neural Networks Based on Robust and Epitaxial Film Memristors

**DOI:** 10.1002/advs.202411925

**Published:** 2025-01-04

**Authors:** Zhenqiang Guo, Guojun Duan, Yinxing Zhang, Yong Sun, Weifeng Zhang, Xiaohan Li, Haowan Shi, Pengfei Li, Zhen Zhao, Jikang Xu, Biao Yang, Yousef Faraj, Xiaobing Yan

**Affiliations:** ^1^ College of Physics Science & Technology School of Life Sciences Institute of Life Science and Green Development Key Laboratory of Brain‐Like Neuromorphic Devices and Systems of Hebei Province Hebei University Baoding 071002 China; ^2^ College of Electron and Information Engineering Hebei University Baoding 071002 China; ^3^ School of Natural Sciences University of Chester Chester CH1 4BJ UK; ^4^ Department of Materials Science and Engineering National University of Singapore Singapore 117576 Singapore

**Keywords:** artificial intelligence system, epitaxial film memristors, graph classification tasks, neuromorphic electronics, weighted echo state graph neural networks

## Abstract

Hardware system customized toward the demands of graph neural network learning would promote efficiency and strong temporal processing for graph‐structured data. However, most amorphous/polycrystalline oxides‐based memristors commonly have unstable conductance regulation due to random growth of conductive filaments. And graph neural networks based on robust and epitaxial film memristors can especially improve energy efficiency due to their high endurance and ultra‐low power consumption. Here, robust and epitaxial Gd: HfO2‐based film memristors are reported and construct a weighted echo state graph neural network (WESGNN). Benefiting from the optimized epitaxial films, the high switching speed (20 ns), low energy consumption (2.07 fJ), multi‐value storage (4 bits), and high endurance (10^9^) outperform most memristors. Notably, thanks to the appropriately dispersed conductance distribution (standard deviation = 7.68 nS), the WESGNN finely regulates the relative weights of input nodes and recursive matrix to realize state‐of‐the‐art performance using the MUTAG and COLLAB datasets for graph classification tasks. Overall, robust and epitaxial film memristors offer nanoscale scalability, high reliability, and low energy consumption, making them energy‐efficient hardware solutions for graph learning applications.

## Graphical abstract

We propose robust and epitaxial Gd: HfO2 film memristors with the SrTiO_3_ buffer layer and develop a novel approach combining array‐algorithm to accelerate graph learning utilizing the weighted echo state graph neural networks (WESGNN). WESGNN employs iterative recursive stochastic mapping for input projection and recursive embedding, thereby nonlinearly transforming input information into a high‐dimensional distinguishable space for analysis.

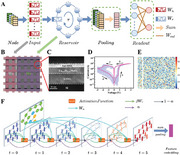

## 1 Introduction

In the era of Artificial Intelligence (AI), the booming development of smart devices and the Internet of Things (IoT) has underscored the importance of graph learning and mining for network management.^[^ ^1^, ^2^, ^3^ ^]^ Graph Neural Networks (GNNs) have demonstrated strong capabilities in capturing graph structure and node features, especially in drug discovery, clustering analysis, and user recommendation applications.^[^ ^4^, ^5^, ^6^ ^]^ However, hundreds of millions of unstructured graph data information per second pose serious challenges to the complementary metal oxide semiconductor (CMOS)‐based hardware processing systems and deep learning computational models, due to the exploding information size and complexity, as well as the urgent need for energy‐efficient, edge‐side portable processing.^[^ ^7^, ^8^, ^9^, ^10^ ^]^

The traditional hardware of von Neumann computing paradigm faces issues due to the physical separation of processing and storage units resulting in large physical size. Additionally, the massive data transmission incurs tremendous energy consumption and longer time delays, severely hampering the operation of AI systems deployed in real‐world scenarios.^[^ ^11^, ^12^, ^13^ ^]^ Moreover, the continual downsizing of transistor nodes, which is increasingly nearing size limits, may slow down Moore's Law and become more uneconomical. Notably, the expensive computational cost of training graph neural networks via error backpropagation is further exacerbated by the addition of tedious nodes and graph embeddings, making real‐time edge processing complexity insatiable.^[^ ^14^ ^]^ In the present landscape, emerging bio‐inspired computational architectures with direct processing of graph data offer novel breakthroughs. They utilize revolutionary in‐memory computing architectures to address increasing hardware challenges and software loads.

The rapidly evolving of in‐memory computing learning paradigm, coupled with neuromorphic devices, is poised to offer promising solutions to the aforementioned challenges.^[^ ^15^, ^16^ ^]^ By simulating the synaptic behaviors of the human brain's nervous system, this approach enables swift handling of massive data through parallel computing while conserving energy.^[^ ^17^ ^]^ Recently, two‐terminal memristor devices have garnered significant attention due to their excellent scalability, low energy consumption, and rapid switching speed.^[^ ^18^, ^19^ ^]^ These memristors exhibit superior characteristics of artificial synapses, including plasticity, learning, and memory, as have been reported in neuromorphic computing.^[^ ^20^ ^]^ Importantly, they avoid data shuttling in many tasks (e.g., gesture classification, heart rate recognition, chaotic pattern prediction, and others), thereby enhancing both time and energy efficiency.^[^ ^21^, ^22^, ^23^, ^24^ ^]^ The cross arrays of memristor devices conveniently store synaptic weights in identical positions, and utilize Ohm's law and Kirchhoff's law to perform multiplication and summation operations in parallel within a single time step, which are typically the most costly and frequent operations in graph neural network training.^[^ ^25^ ^]^ However, current applications of amorphous/polycrystalline oxide‐based memristors suffer from challenges such as uncontrollable resistive state, considerable high variability, and additional energy/time requirements.^[^ ^22^ ^]^ These limitations could significantly undermine the reliability and effectiveness of in‐memory GNN systems. Wang employed amorphous TiN/Ta/TaO_x_/TaN random resistive memory cells in echo state GNN learning, revealing the endurance and switching energy require further improvement.^[^ ^14^ ^]^ Epitaxial film memristors have few grain boundaries and defects, which is likely more conducive to oxygen vacancy migration and controllable resistance switching.^[^ ^26^, ^27^ ^]^ Therefore, the development of memristors with enhanced endurance, power efficiency, and reliability is imperative.

In this study, we systematically present robust and epitaxial Gd:HfO_2_ film memristors with the SrTiO_3_ buffer layer and develop a novel approach combining array‐algorithm to accelerate graph learning utilizing the weighted echo state graph neural networks (WESGNN). The memristors exhibit impressive characteristics including high switching speed (20 ns), low energy consumption (2.07 fJ), multi‐value storage (4 bits), high endurance (10^9^), and powerful capability to simulate biological synapses. These include long‐term potentiation/depression (LTP/LTD), paired‐pulse facilitation/depression (PPF/PPD), post‐tetanic potentiation (PTP), spiking time‐dependent plasticity (STDP), spiking rate dependent plasticity (SRDP), spiking duration dependent plasticity (SDDP), spiking amplitude dependent plasticity (SADP), and Bienenstock−Cooper−Munro (BCM) rules. Furthermore, benefiting from appropriately dispersed conductance distribution (standard deviation = 7.68 nS) of the memristors, we leverage the randomness of set voltage in resistive switching to physically realize the random weight matrix of the WESGNN. This approach not only minimizes hardware cost and ensures exceptionally high reliability but also facilitates energy‐efficient memory computing. Notably, the weighted echo state network employs iterative recursive stochastic mapping for input projection and recursive embedding, thereby nonlinearly transforming input information into a high‐dimensional distinguishable space for analysis. This overcomes the laborious training process of traditional graphical neural networks while finely regulating the relative weights of input nodes and recursive features, achieving efficient and cost‐effective graph learning. We achieve superior graph classification on the two representative MUTAG and COLLAB datasets, achieving the highest accuracy up to 94.15% and 74.03%, respectively. This study presents a promising approach for utilizing robust and epitaxial film memristors in next generation graph computing hardware.

## 2 Results and Discussion

### 2.1 Weighted Echo State Graph Neural Network Based on Memristors

**Figure 1:**
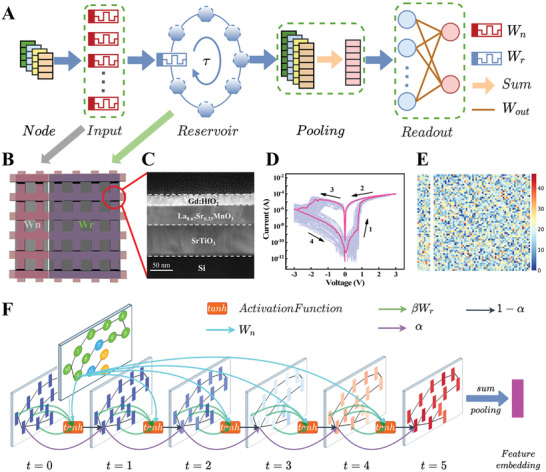
The graph computing system with integrated design of array and algorithm. A)The architecture diagram of the WESGNN with memristor arrays. B) The schematic diagram of memristor arrays, the left section demonstrating the node weight matrix *W_n_*, the right section demonstrating the recursive weight matrix *W_r_*. C) The cross‐sectional TEM of the Gd:HfO_2_ memristor device. D) The *I–V* characteristics of the device for fifty cycles. E) The experimental conductance matrices corresponding to the above two parts respectively. F) Schematic of the forward propagation process in the echo layer of WESGNN.

**Figure** **1** illustrates a novel array‐algorithm codesign of WESGNN based on Gd:HfO_2_ memristors for energy‐efficient graph learning. On the array side, the stochastic distribution of set voltages provides a promising source of randomness for generating large‐scale, low‐power, and fast memristor arrays.^[^ ^28^, ^29^, ^30^ ^]^ On the algorithm side, the architecture of WESGNN incorporates an echo state network comprising an input layer, echo layer, and readout layer.^[^ ^14^, ^31^, ^32^, ^33^ ^]^ Here the graph‐structured data undergo gradual transformation into more efficient feature representations through consecutive untrained nonlinear random projections.^[^ ^34^ ^]^ As depicted in Figure 1A, the system operation procedure can be roughly divided into three steps. First, all node features of the graph‐structured data are loaded into the input layer. Then the feature representation results obtained in the previous time step are fed into the connected echo layer, where the connection weights of the input and echo layer are utilized using random conductance values from the memristor arrays. The network is iteratively updated to achieve optimal feature representations through non‐linear mapping of the input information to a high‐dimensional space. Subsequently, features mapped from the high‐dimensional space undergo summation pooling operation to obtain the total features of this data, and the classification result is returned by the readout layer. This hardware‐software synergy fully utilizes the advantage of memristor arrays, including easily accessible randomness and low‐power operation, thereby providing low‐cost and easily stackable stochastic memristor arrays for neuromorphic computing. During training, the connection weights of the input and the echo layers are fixed and the simple ridge regression method is used to optimize the network connection weights of the readout layer. This simplifies the training problem of the network into a regression problem, minimizing complexity and training for WESGNN.^[^ ^34^, ^35^ ^]^ Crucially, in the forward propagation process, WESGNN accurately captures key information by adjusting the relative importance of the same node itself and neighboring nodes, thereby improving graph classification accuracy (refer to Figures S1 and S2 (Supporting Information) for specific details of the forward propagation process).

As depicted in Figure 1B, the weight matrices of the input and echo layers in WESGNN are physically mapped onto two Gd:HfO_2_ memristor subarrays. Here, the node conductance matrix *W_n_* ∈ *H* × (*u* + 1)adjusts the influence of the node input feature information on the node's internal state, while the recursive conductance matrix*W_r_* ∈ *H* × *h*regulates the impact of neighboring nodes on the specified node's internal state. Figure 1C illustrates a transmission electron micrograph (TEM) cross‐section diagram of the Gd:HfO_2_ device with a clear interface. The simulated circuit‐level architecture design based on Gd:HfO_2_ memristor arrays is shown in Figure S3 (Supporting Information). Figure 1D illustrates the fifty‐cycle current–voltage (*I–V*) characteristics, revealing apparent programming randomness in the statistical results of set/reset voltages and high/low resistance state (HRS/LRS) distributions (Figure S4, Supporting Information). We utilize National Institute of Standards and Technology (NIST) measurements to demonstrate that the conductance values of the memristor arrays are random (Table. S1, Supporting Information). The shape of the *I–V* curve is typical of filamentary‐type resistive switching, namely the sharp current steps associated with filament formation/dissolution.^[^ ^36^ ^]^ Due to the trivalent Gd ion replacing the tetravalent hafnium in the crystal, oxygen vacancies are present in the oxide layer.^[^ ^37^ ^]^ Few grain boundaries in epitaxial Gd:HfO_2_ film offer a high‐speed migration pathway for oxygen vacancy transport and help stabilize conductive filaments.^[^ ^26^, ^27^ ^]^ Figure 1E displays the conductance diagrams of *W_n_* and *W_r_*. All devices within the prepared memristor arrays are biased to their average set voltage. If the applied voltage exceeds their set voltages, a few devices undergo the set process, thereby shaping the stochastic memristor array. Figure 1F illustrates the detailed forward propagation process, where the network structure of the echo layer utilizes a recurrent neural network. The nodes of the graph‐structured data are represented as ${\left\{x_{i}\right\}}_{i=1}^{n},x_{i}\in R^{d}$. Taking node **x**_*i*_ as an example, it undergoes a stochastic mapping in the node matrix, resulting in its internal feature representation $u_{i}=W_{n}x_{i}\in R^{h}$ in the input layer. Subsequently, in the echo layer, the feature representation of node **x**_*i*_is initialized as $s_{i}^{\left(0\right)}=0\in R^{h}$. In every subsequent iteration, $s_{i}^{\left(t+1\right)}$ is composed of the node matrix **u**_*i*_, the state of the previous time step $s_{i}^{\left(t\right)}$, and the feature representation of all the neighboring nodes processed by a stochastic mapping in the recursive matrix $\left\{s_{k}^{\left(t\right)}\right\},k\in N\left(i\right)$(where*N*(*i*) indicates the collection of neighboring nodes of the node **x**_*i*_) (see Experimental Section for the detailed computing procedure). This continuous nonlinear stochastic mapping of $s_{i}^{\left(0\right)}$ can confer **x**_*i*_a distinctive feature representation $s_{i}=s_{i}^{\left(5\right)}$. The feature representations ${\left\{s_{i}\right\}}_{i=1}^{n}$ of all nodes are summed to obtain the resulting feature representation $e=\sum_{i=1}^{n}s_{i}$ of the graph‐structured data.

### 2.2 Structural Characterizations

**Figure 2:**
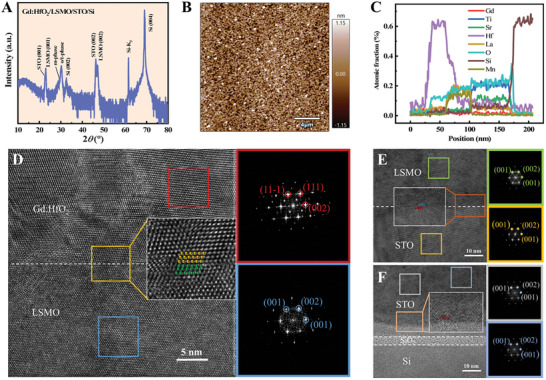
The characterization images of epitaxially grown Gd:HfO_2_/LSMO/STO/Si structure thin films. A) The XRD results of Gd:HfO_2_ thin films. B) The AFM image of the film at 20 × 20 µm region. C) The EDS of Gd, Ti, Sr, Hf, La, O, Si, Mn elements in the Gd:HfO_2_ based device. D) The HRTEM image of Gd:HfO_2_ grown on LSMO layer and the FFT of the selected regions. E) The HRTEM image of the LSMO layer grown on the STO layer and the FFT of the STO and LSMO layers. F) The HRTEM of STO layer and the FFT results.

During the growth process of epitaxial Gd:HfO_2_ films, parameters such as temperature, O_2_ pressure, and laser frequency significantly influence the thermodynamics and crystallization of films. Subsequently, we adjust the growth process in order to obtain films with high‐quality films under optimal conditions (700 °C, 20 Pa, 2 Hz). The X‐ray diffraction (XRD) results of Gd:HfO_2_/LSMO/STO/Si structure films are presented in **Figure** **2**A. The diffraction peaks within the range of 27–32° correspond to the monoclinic phase (m‐phase) and orthorhombic/tetragonal phase (o/t‐phase), respectively. An atomic force microscope (AFM) was used to scan the morphology in the 20 × 20 µm region, revealing relatively smooth surfaces of the thin films, as shown in Figure 2B. Energy dispersive X‐ray spectroscopy (EDS) and elemental mapping images were used to characterize the element distribution, as shown in Figure 2C and Figure S6 (Supporting Information). Further examination of crystallinity and nanoscale structure was conducted using high‐resolution transmission electron microscopy (HRTEM) images of the Gd:HfO_2_/La_0.67_Sr_0.33_MnO_3_(LSMO)/SiTiO_3_(STO)/Si heterostructure. Figure 2D illustrates the epitaxial structure of the Gd:HfO_2_ thin films. The magnified image of the yellow‐marked region shows a clear interface of Gd:HfO_2_/LSMO, suggesting the potential role of this interfacial layer in stabilizing epitaxial structure. A measurement of d_111_ = 2.95 Å was obtained from the Fast Fourier transform (FFT) of the Gd:HfO_2_ thin films marked in the red squares. Additionally, the FFT of the LSMO region is marked in the blue squares. Similarly, Figure 2E,F shows the HRTEM and FFT images of the STO layer and the LSMO layer, indicating that both layers grow tightly bonded along the (001) direction, demonstrating good epitaxial characteristics. These results demonstrate the great potential of epitaxial Gd:HfO_2_ films for the construction of high‐performance memristors and their neuromorphic computing.

### 2.3 Robust Switching Performance and Synaptic Functions

**Figure 3:**
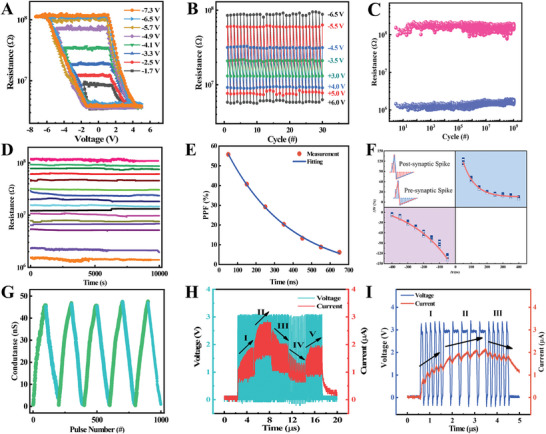
The Resistive switching characteristics of robust and epitaxial Gd:HfO_2_ based device. A) Multiple *R−V* hysteresis loops at different pulse amplitude. B) The Resistance switching with different resistance states. C) The endurance performance test of the device. D) Retention properties of 4‐bit resistance states. E) PPF index as a function of Δt. F) STDP curve between pre‐synaptic neuron and post‐synaptic neuron under the Hebbian learning rule. G) The repeatability of LTP and LTD characteristics by 100 consecutive positive and negative pulses. The synaptic weight changes under a set of pulse trains with the different H) frequency sequence, and I) duration sequence.

To investigate the gradual conductance switching properties and multistate programming of the Gd:HfO_2_ memristor, we analyzed the hysteresis characteristics and cyclability of the resistance versus voltage. Under the 0V → negative maximal voltage (V_max‐_) → positive maximal voltage (V_max+_) → 0 V pulse voltage scanning sequence (Figure S7A, Supporting Information), we continuously could manipulate the memristor to various intermediate resistive states by varying *V*_max‐_ (−1.7, −2.5, −3.3, −4.1, −4.9, −5.7, −6.5 and −7.3 V) with a fixed pulse width 50 ns and V_max+_ (+5.5 V) (**Figure** **3**A). In the cyclic voltage sequence measurement (Figure S7B, Supporting Information), reversible switching of Gd:HfO_2_ memristor was achieved by applying different pulse voltage with a fixed pulse width of 50 ns (Figure 3B). The continuous and multilevel tuning of the conductance state by the nonvolatile switching of the Gd:HfO_2_ memristor at nanosecond rate not only accurately simulates the plastic change of synaptic weights but also reduces time and power consumption during training the neuromorphic computing systems.^[^ ^38^ ^]^ Endurance and retention capabilities are two important characteristics of the memristors. The robust Gd:HfO_2_ memristor exhibits a remarkable endurance, as depicted in Figure 3C, with a cycle endurance of 10^9^ cycles, meeting the device requirements of running WESGNN. This high endurance can be attributed to the high‐quality epitaxial films with few grain boundaries. Additionally, after applying ten consecutive positive or negative direct current (DC) sweeps to the device, the current exhibits a clear tendency for increasing or decreasing, as shown in Figure S8 (Supporting Information). Figure 3D shows the retention time of the 4‐bit memristor storage state, which remains stable for up to 10^4^ seconds without significant degradation. This demonstrates the strong potential and robustness of the epitaxial Gd:HfO_2_ memristor for practical storage applications.

Synaptic plasticity, fundamental for information processing and neural computing, can be achieved based on switching dynamics. By varying pulse parameters, synaptic weights can be updated, allowing implementation of functions in artificial synapses. PPF is a typical synaptic behavior in neurological studies, which refers to conductance response caused by the second pulse is greater than that of the first pulse. The effect of PPF on the change of synaptic weight can be studied by changing the pulse interval between two consecutive pulses. As shown in Figure 3E, the smaller the pulse interval, the greater the conductivity change, and the PPF index is fitted by an exponential function $PPF=\frac{G_{2}-G_{1}}{G_{1}}\times 100\%=C_{1}\times e^{\frac{-t}{\tau_{1}}}+C_{2}\times e^{\frac{-t}{\tau_{2}}}$ where G_1_ and G_2_ are the conductance after the first and second pulses. The memory effect can be enhanced by reducing the pulse interval, which is consistent with the rules of biological synaptic learning. Intrinsic conduction dynamics cause the synaptic behavior. The resistance mechanism is due to the oxygen vacancy filament switching. The electric field formed by applying a forward pulse causes some oxygen vacancy migration to form conductive channels. For PPF, a single short pulse does not excite enough oxygen vacancies to form a complete conduction channel between the two electrodes, and if a subsequent pulse causes more oxygen vacancies to enter the gap between the electrodes, it results in the gradual increase of the device conductance, similar to the PPF phenomenon in biological synapses. For PPD, the electric field formed by applying the opposite pulse (or decreasing the forward pulse) or the spontaneous diffusion of the oxygen vacancy will destroy the conductive channel, resulting in the gradual reduction of the device conductance, similar to the phenomenon of PPD in biological synapses. STDP is one of the important Hebbian synaptic learning rules. As is shown in Figure 3F, ΔW is modulated by a pair of electrical spikes applied to pre‐synaptic neuron and post‐synaptic neuron. When Δt > 0, the neural stimulation signals of the pre‐synaptic neuron play a promoting role in the production process of that of the post‐synaptic neuron, and the synaptic weight increases. When Δt < 0, the neuronal stimulation signals of pre‐synaptic neurons inhibit that of the post‐synaptic neuron, and the synaptic weight decreases. The STDP curve if fitted by $\Delta W=\left\{\frac{A\times \exp\left(\Delta t/\tau_{0}\right),\Delta t>0}{-A\times \exp\left(\Delta t/\tau_{0}\right),\Delta t<0}\right.$ where A is proportional coefficient, τ_0_ is the time coefficient, and Δt is the pulse interval. This indicates memristor can simulate the temporal information processing capabilities of biological synapses.

The gradual controllable property of conductance in Gd:HfO_2_ memristor enables the implementation of LTP and LTD functions. A variable pulse amplitude programming scheme is particularly conducive to improving the dynamic range and linearity rate of conductance, as illustrated in Figure 3G. LTP and LTD are key mechanisms in neuromorphic computing, essential for the development of advanced intelligent systems with learning and memory functions.^[^ ^39^ ^]^ The conductance change of the device is strongly influenced by the pulse parameters applied, such as pulse amplitude, width, and interval (Figure S9, Supporting Information). Various synaptic plasticity rules, including PPD, PTP, other STDP, were simulated subsequently (Figures S10 and S11, Supporting Information).^[^ ^40^, ^41^ ^]^ Notably, during the simulation of the SRDP rule, pulses with same frequency can trigger opposite weight change, depending on the historical experience of the synapse. As shown in Figure 3H, pulse stimuli with relatively high frequency in the first and second parts increase the conductance of the device, while those with relatively low frequency in the third and fourth parts obtain low conductance. With the higher frequency of pulse stimulus in the fifth part compared to the fourth part, the conductance of the device increases accordingly. It is worth noting that the first and third parts have the same frequency of pulse stimulus but induce opposite conductance changes, as the third part is not sufficient to counteract the decay of conductance after the second part. Similarly, the relatively low‐frequency pulse stimulus in the fourth part decreases the conductance, resulting in the fifth part inducing an opposite conductance change compared to the third part. The results suggest that the conductance in the fifth part appears slightly larger than in the first part, indicating the memory characteristics of the device. Additionally, SDDP, SADP, BCM, and learning‐forgetting‐relearning functions are also successfully mimicked in the Gd:HfO_2_ memristor, as shown in Figure 3I and Figures S12−S14 (Supporting Information).^[^ ^41^ ^]^ Table S2 (Supporting Information) compares the performance of the related device in recent years, fully justifying the unrivaled advantages of robust and epitaxial Gd:HfO_2_ memristor.

### 2.4 Graph Classification with Weighted Echo State Graph Neural Network

To prove the powerful performance of WESGNN based on robust and epitaxial Gd:HfO_2_ memristors in graph classification tasks, experiments were conducted on two types of datasets: the molecular dataset MUTAG and the social network dataset COLLAB.^[^ ^42^, ^43^ ^]^ On the one hand, the MUTAG dataset comprises 188 graphs, where each graph represents a nitro compound, with nodes representing atoms and edges representing chemical bonds. The dataset is categorized into positive and negative classes based on their mutagenic effect on bacteria. On the other hand, the COLLAB dataset comprises 5000 graphs, where each graph represents a network of relationships among researchers. Nodes denote researchers and their collaborators, while edges represent collaborative relationships between researchers. The dataset is classified into three categories based on research fields, namely high energy physics (HE), condensed matter physics (CM), and astrophysics (AP). In WESGNN, the randomness of the set voltage in resistive switching is employed to achieve a random and fixed physical projection of the echo layer. This approach significantly reduces the computational burden associated with training the output layer alone. Particularly, the appropriately dispersed conductance distribution (standard deviation = 7.68 nS) and precise regulation of the relative weights of input nodes and recursive features are employed to achieve superior graph classification performance on the MUTAG and COLLAB datasets.

**Figure 4:**
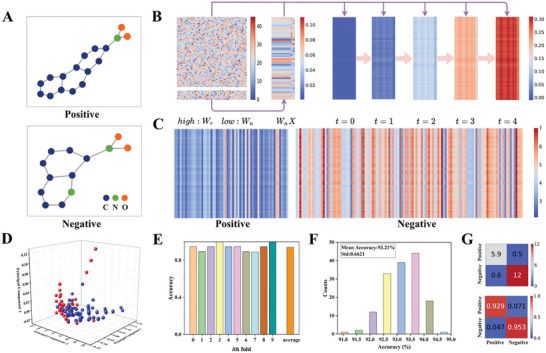
Classification of the MUTAG dataset. A) The schematic diagram of the graph samples. B) The iterative process for graph vector feature representation. C) The graph vector feature representations for two classes of the MUTAG dataset. D) Mapping the graph vector feature representation to 3D space using PCA. E) The accuracy for each fold in the tenfold cross‐validation. F) The accuracy distribution of 150 experiments. G) The confusion matrix for average classification results. Normalizing the above matrix obtains the following matrix.

**Figure** **4**A shows examples of molecular structures from the MUTAG dataset for the positive and negative classes, with different colored nodes representing different atoms (Additional molecular structures can be found in Figure S15, Supporting Information). Figure 4B shows the visualization results of recursive weight matrix*W_r_*, node weight matrix*W_n_*, feature weight matrix*W_n_X*_,_ and the feature representations of the nodes at various iterative time steps during the forward propagation process. In order to prevent the over‐smoothing issue resulting from excessive network layers,^[^ ^44^ ^]^ the embedding layer of WESGNN iterates cyclically five‐time steps. As the time step (t) increases, the feature representations of the nodes capture more topological information, and each feature representation of each node increases at different rates. When t = 4, the feature representations of all nodes are summed and pooled to obtain the feature representation of the graph. Figure 4C shows the visualization of feature representations obtained for all graphs in the MUTAG dataset. The positive class comprises 63 graphs, and the negative class comprises 125 graphs. Most graph feature representations in the positive class exhibit low values, while those in the negative class display higher values. This indicates that WESGNN can obtain graph feature representations with discriminative information, ensuring dissimilar feature representations among different class samples.

To demonstrate that the final feature representations obtained from the graph data are linearly separable in high‐dimensional space, principal component analysis (PCA) was employed to map the graph feature representations to 3D space. This visualization illustrates the distribution of the graph feature representations (see Figure S16, Supporting Information for the distribution of the feature representations obtained from the graph data in two‐dimensional space), with blue and red colors denoting representations from the positive and negative classes, respectively. Figure 4D shows that the majority of graph feature representations can be linearly separable by a simple linear classifier. Additionally, to mitigate potential biases resulting from a single division between training and validation sets, tenfold cross‐validation was utilized to evaluate the classification performance of the MUTAG dataset, and the per‐fold accuracy is shown in Figure 4E, with an average classification accuracy of ≈94.15%. Figure 4F shows the accuracy distribution of WESGNN for 150 experiments on the MUTAG dataset, with an average classification accuracy of ≈ 93.21%. This performance is comparable to state‐of‐the‐art deep learning algorithms such as QS‐CNNs (93.13%)^[^ ^45^ ^]^ and Graph‐JEPA (91.25%).^[^ ^46^ ^]^ Notably, the standard deviation is ≈0.66, indicating relatively stable calcification performance (Figure S17, Supporting Information demonstrates the effect of hyperparameters). Figure 4G displays the confusion matrix of prediction results, indicating that on average, 5.9 out of every 6.4 positive categories of graph data, and 12 out of every 12.6 negative categories of graph data were correctly categorized (The confusion matrix for all classification results in the tenfold cross‐validation is shown in Figure S18, Supporting Information).

**Figure 5:**
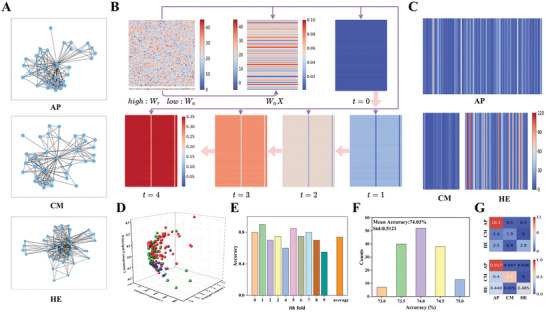
Classification of the COLLAB dataset. A) The graph examples for different classes. B) The iterative process for graph feature representation. C) The graph vector feature representations for three classes of the COLLAB dataset. D) The graph vector feature representation mapped to 3D space by PCA. E) The accuracy of each fold in the tenfold cross‐validation. F) The accuracy distribution of 150 experiments. G) The confusion matrix for average classification results. Normalizing the above matrix obtains the following matrix.

**Figure** **5**A shows examples of researchers’ ego network structures in the COLLAB dataset for astrophysics, condensed matter physics, and high energy physics, respectively (see Figure S19, Supporting Information for additional social network structures). Each node represents a researcher, and nodes within the same ego network have the same color. For learning purposes, 200 graphs were randomly selected from the COLLAB dataset. Figure 5B shows the visualization of weights and feature representations. *W_n_* has a dimension of 2 × *h* (2 contains one‐dimensional node input features and one‐dimensional bias). As all nodes have the same input features, each node gets the same internal node features after the projection of *W_n_*. Similar to the MUTAG dataset, the embedding layer is cycled for 5 iterations. During the forward propagation, as the number of iterations increases, the feature representation of each node continuously aggregates feature information contained in neighboring nodes from the previous time step, resulting in different node feature representations. Figure 5C presents the visualization of various types of final graph feature representations in the COLLAB dataset. In this case, most of the graph feature values in condensed matter physics are relatively low, on the contrary, whereas in high energy physics, most graph feature values are relatively high. This discrepancy is attributed to the substantial difference in the topological structure of graphs between condensed matter physics and high energy physics, enabling easy distinction between them.

Figure 5D depicts the visualization of graph feature representations mapped into three dimensions using PCA analysis (see Figure S20, Supporting Information for the distribution of feature representations obtained from the graph data in two dimensions). The green and blue colors are linearly separable, whereas the red color partially overlaps with the other two colors. This overlap is attributed to the existence of similar topological structures of graphs in astrophysics as in the other two categories (condensed matter physics and high energy physics). Utilizing a simple readout layer to obtain prediction results from the graph feature representations, Figure 5E shows the accuracy for ten fold cross‐validations, yielding an average classification accuracy of 74.03%. Notably, when the 2nd‐fold is used as the test set, most graphs in the test set exhibit dissimilar topologies with high prediction accuracies, demonstrating the capability of WESGNN to predict graph data in condensed matter physics and high energy physics effectively. Figure 5F shows the accuracy distribution of WESGNN across 150 experiments on the COLLAB dataset (Figure S21, Supporting Information demonstrates the effect of hyperparameters), with an average classification accuracy of ≈74.03% and a standard deviation of ≈0.51. This performance is comparable to or better than deep learning algorithms such as DiffWire (72.24%)^[^ ^47^ ^]^ and GraphSAGE (73.90%).^[^ ^48^ ^]^ Figure 5G shows the confusion matrix of prediction results, revealing that over 40% of graphs in condensed matter physics (high energy physics) are misclassified as astrophysics, while condensed matter physics (high energy physics) is almost rarely misclassified as high energy physics (condensed matter physics) (the confusion matrix of all classification results in the tenfold cross‐validation is shown in Figure S22, Supporting Information). This highlights the importance of the topological structure of the graph in determining the quality of classification results, particularly when the intrinsic feature representations of all nodes are the same (which employs tenfold cross‐validation with nine folds for training and one fold for testing). In summary, the optimal classification accuracy achieved by WESGNN can be attributed to the conductance of Gd:HfO_2_ memristors which follow a Gaussian distribution with a suitable degree of dispersion (Figure S23, Supporting Information).

## 3 Conclusion

In conclusion, we have obtained robust and epitaxial Gd:HfO_2_ film memristors. And we also demonstrated the effectiveness of the WESGNN system with Gd:HfO_2_ memristors for graph learning tasks. The device achieves high switching speed (20 ns), low power consumption (2.07 fJ), multi‐value storage (4 bits), high endurance (10^9^) while simulating various synaptic functions. Additionally, the utilization of appropriately dispersed conductance distribution (standard deviation = 7.68 nS) and the randomness in resistive switching, enhancing the robustness, cost‐effectiveness, and scalability of the system, making it an excellent stochastic fundamental block for stochastic computing. Notably, the physical random conductance mapping facilitates feature representation, circumventing the need for cumbersome back‐propagation optimization typical in traditional graph neural networks. Furthermore, by dynamically adjusting the relative importance of nodes and edges in the graph data, our system maximizes recognition accuracy. The collaborative integration of array and algorithm enables efficient parallel processing for complex graph data, leading to high recognition rates of 94.15% and 74.03% in the MUTAG and COLLAB datasets, respectively. Therefore, the high‐performance and epitaxial Gd:HfO_2_ memristors contribute to the reliability and low‐power operation of the system, positioning it as a promising platform for graph information processing. We anticipate that this study will inspire further exploration and development of integrated software and hardware systems for various neuromorphic computing applications, driving advancements in the field.

## 4 Experimental Section

### Device Fabrication

The epitaxial Gd:HfO_2_ film memristors were prepared by pulsed laser deposition. Initially, heavily doped p‐type silicon was used as the substrate. The silicon substrates were soaked in acetone and ethanol for 5 min at room temperature, followed by a treatment in a solution of deionized water and hydrofluoric acid diluted at a ratio of 3:1 for 90 s. Subsequently, the substrates were then ultrasonically cleaned in deionized water for 10 min, and the substrates were then dried using pure nitrogen. The STO films were deposited at 700 °C/1 Pa; the LSMO films were deposited at 700 °C/26 Pa. The deposition frequency was maintained at 5 Hz. Then, the Gd:HfO_2_ films were deposited at 700 °C/20 Pa/2 Hz. Throughout the deposition process, an oxygen atmosphere was maintained to facilitate in situ annealing. Finally, top palladium electrodes with a diameter of 50 µm were grown using a mask plate in an argon atmosphere at 1 Pa with a flow rate of 25‐sccm by DC magnetron sputtering. This comprehensive fabrication process ensured the precise formation of epitaxial Gd: HfO2‐based film memristors, meeting the required specifications for subsequent experimental investigations and applications.

### Structural and Electrical Measurements

The XRD spectra were obtained using a diffractometer. High‐resolution transmission electron microscopy (HRTEM), energy‐dispersive X‐ray spectroscopy (EDS), and elemental mapping images were acquired using a Fei Tecnai G2 F20 ST FE‐TEM instrument. A commercial AFM system (Cipher‐S, Asylum Research, GAI‐SA) was employed to observe the surface morphology of the samples. These analytical techniques enabled comprehensive characterization of the Gd: HfO2‐based film memristors, providing valuable insights into their structural, compositional, and morphological properties. The electrical properties were tested by using the Keithley 4200 system.

### Graph Classification Demonstration

The echo state network only needs to train the connection weights of the readout layer, which can greatly reduce the training time. To ensure that the echo state network algorithm operates effectively, the reservoir layer must possess the echo state property. This was achieved when the activation function, typically tanh, was applied, and the spectral radius of the weight matrix was less than 1.^[^ ^49^ ^]^ Therefore, the node weight matrix*W_n_* and recursive weight matrix*W_r_* were generated as scaled random conductance expressed by the equation: $W_{n}=\alpha_{n}C_{n}\in R^{h\times (d+1)}$, $W_{r}=\alpha_{r}C_{r}\in R^{h\times h}$, whereα_*n*_andα_*r*_are scaling factors,*C_n_* and*C_r_* are the random conductance of the input and echo layer, respectively, and *h* and *d* are denoted as the number of neurons in the echo layer and the dimension of the input nodes, respectively.

Given a graph*G* = (*V*, *E*), where $V={\left\{x_{i}\right\}}_{i=1}^{n}$ is the set of nodes in*G*,*E* = {(*u*, *v*)|*u* ∈ *V*, *v* ∈ *V*}is the set of edges in *G*, and n is the number of nodes in*G*, $x_{i}\in R^{d+1}$ (d‐dimensional feature representation and 1‐dimensional bias) is the input feature vector of the node, and the input feature vectors of graph nodes in MUTAG and COLLAB are 8 and 2 dimensions, respectively. The internal feature representation **u**_*i*_ of node is obtained by applying **x**_*i*_ through the input layer*W_n_*, which is expressed by equation: $u_{i}=W_{n}x_{i}\in R^{h}$, Where $u_{i}\in R^{h}$. Then the node obtains its feature representation through the echo layer. Defining the feature representation of node **x**_*i*_ at t step as $s_{i}^{\left(t\right)}$, the set of neighboring nodes of **x**_*i*_ as*N*(*i*), and the projected feature vector obtained by applying **x**_*i*_ through *W_r_* at t step as $W_{r}s_{j}^{\left(t\right)}$.

When*t* = 0, the feature representation of the node was initialized as $s_{i}^{\left(0\right)}=0\in R^{h}$. Compared to the feature representation of**x**_*i*_ at t step, the feature representation of **x**_*i*_at t+1 step converges new topological information by adding the node's internal information and the feature information of the neighboring nodes of **x**_*i*_at t step. In addition, the importance of the above two parts of information was adjusted by adding a trade‐off factor. Thus, the feature representation of **x**_*i*_ at t+1 step consists of the feature representation of **x**_*i*_at t step, its intrinsic feature representation **u**_*i*_, and the projected feature vectors of all its neighboring nodes. The equation of forward propagation is expressed as:

(1) $$s_{i}^{\left(t+1\right)}=\alpha s_{i}^{\left(t\right)}+\left(1-\alpha \right)\tanh\left(u_{i}+\beta \sum_{j\in N(i)}W_{r}s_{j}^{\left(t\right)}\right)$$

whereαis the leakage factor, *tanh*is the activation function, andβis the trade‐off parameter between node information and neighboring node information. The forward propagation process of the echo layer is cycled and iterated five‐time steps to obtain the optimal feature representation of all nodes. The optimal feature representations of all nodes are summed and pooled to obtain the optimal feature representation of the graph, which is expressed as: $e=\sum_{i=1}^{n}s_{i}$_._

After applying the graph feature representation through the readout layer, the predicted classification result of the graph was obtained as $\tilde{y}=W_{out}e$. The output results in MUTAG and COLLAB belong to {−1, 1} and {0, 1, 2}, respectively. WESGNN only needs to train the network connection weights *W_out_* of the readout layer, which is a fully connected layer. Specifically, ridge regression was used to obtain *W_out_*. The equation is expressed as: *W_out_* = *y* **e** ^T^(**ee** ^T^ − λ*I*)^−1^, where *y* denotes the true label of the graph, λ is the regularization parameter, and*I*is the unit matrix, as calculated in Figure S24 (Supporting Information).

## Conflict of Interest

The authors declare no conflict of interest.

## Supporting information



Supporting Information

## Data Availability

The data that support the findings of this study are available from the corresponding author upon reasonable request.
