# Supplementary material for: Weighted Echo State Graph Neural Networks Based on Robust and Epitaxial Film Memristors
Source: Adv Sci (Weinh). 2025 Jan 4;12(8):2411925. doi: 10.1002/advs.202411925 (PMC11848613; doi:10.1002/advs.202411925)
Supplement: Supplementary file 1 — Supporting Information [file ADVS-12-2411925-s001.docx]

Supplementary Materials for

**Weighted echo state graph neural networks based on robust and epitaxial film memristors**

Zhenqiang Guo et al.

† Corresponding author. Email: yanxiaobing@ime.ac.cn.


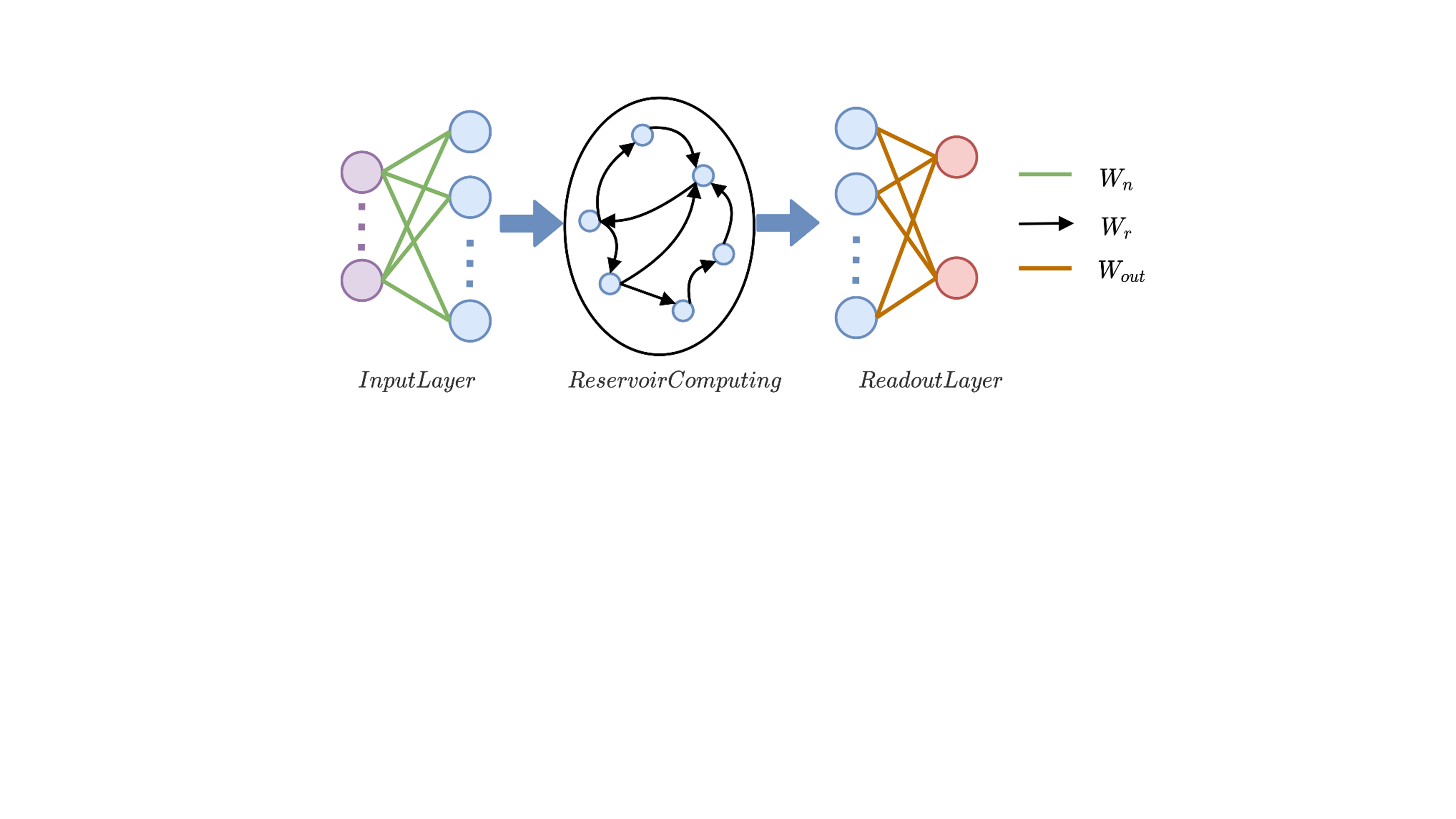


**Fig. S1. The schematic of reservoir computing.**

The network architecture comprises the input layer, the reservoir computing layer, and the readout (or output) layer. The nodes in the input and readout layers are fully connected, while those in the reservoir computing layer are cyclically connected. During the training phase, the network connection weights of the reservoir computing layer remain unchanged after initialization. Only the network connection weights of the readout layer undergo training using a simple regression method, which significantly reduces the training time and complexity of the system.


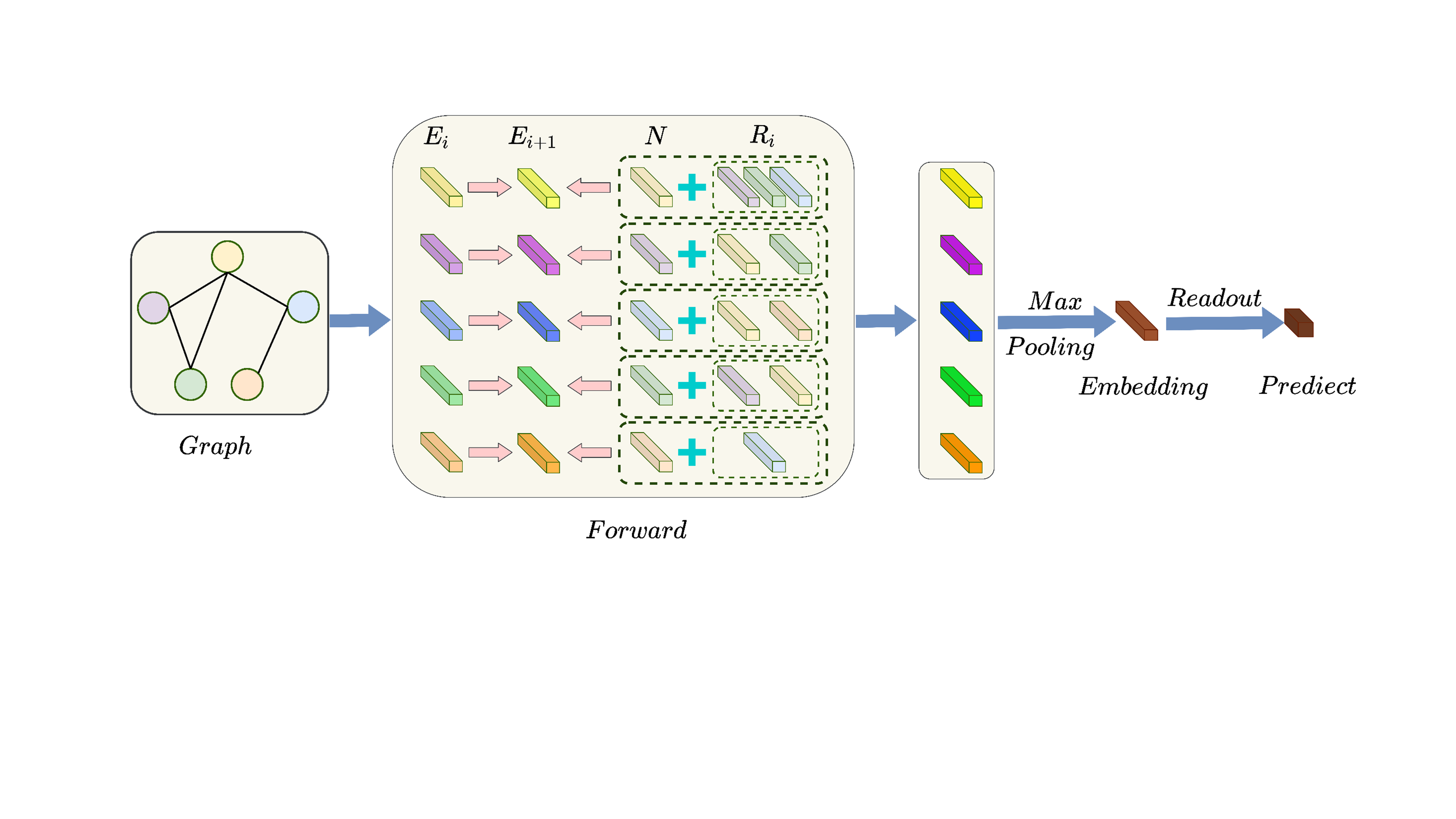


**Fig. S2. The forward propagation process of WESGNN.**

The node feature representation$E_{t+1}$at$t+1$is calculated from the node feature representation $E_{t}$at $t$, the intrinsic feature representation of the node $N$, and the feature representations of all neighboring nodes $R_{t}$at $t$. The weight of $E_{t}$ and the weights of $N$ and $R_{t}$are summed to 1 (the weights are indicated by pink arrows). To enable flexible adjustment of the relative importance of different information sources in node feature representations, different weights are added to$N$and $R_{t}$ (indicated by dashed boxes). The topological information of the graph data is obtained by fusing the node information with that of its neighboring nodes. Finally, the feature representations of the graph data are fed through the readout layer to obtain the classification results.


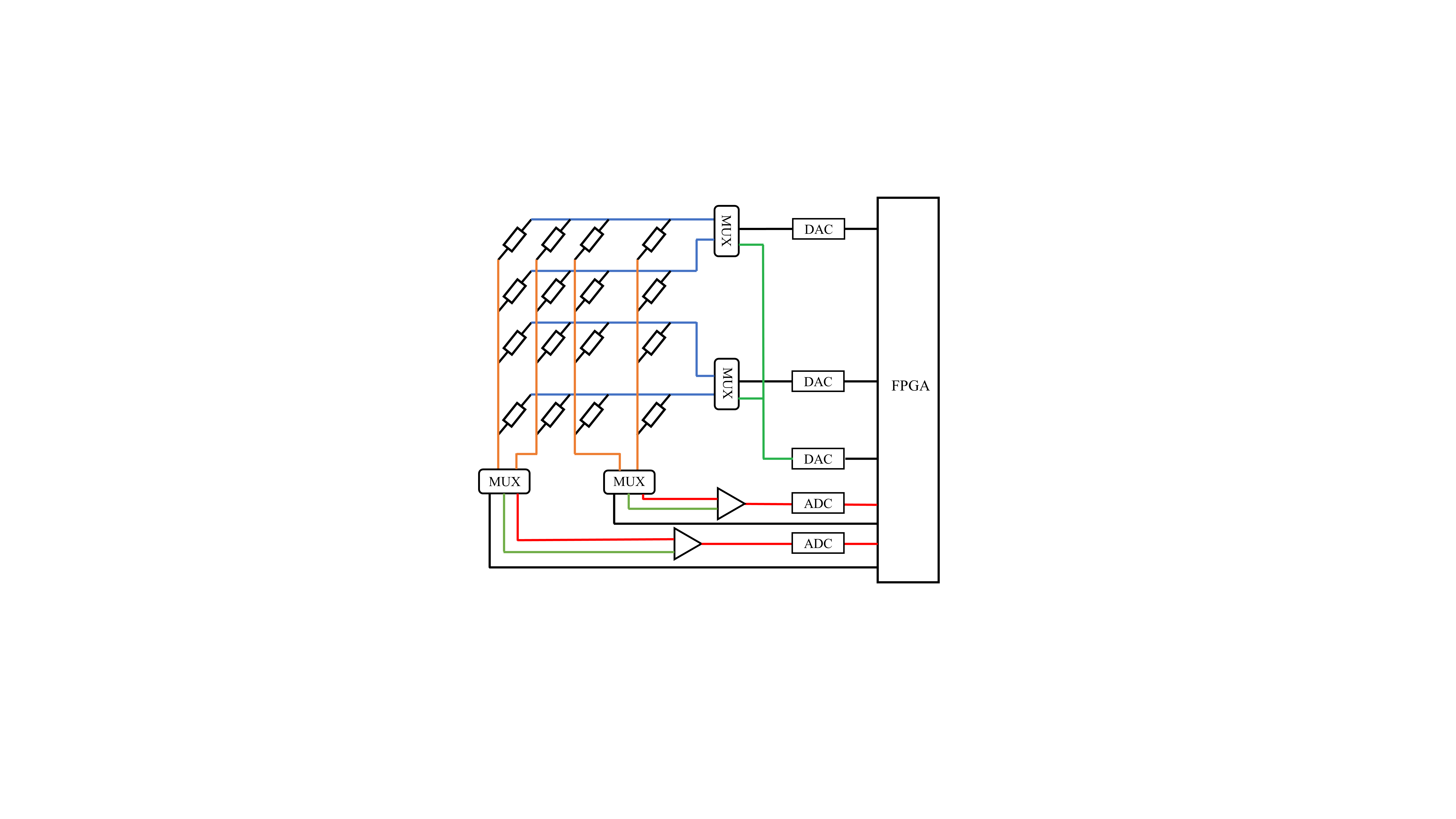


**Fig. S3. The circuit-level architecture based on Gd:HfO_2_ memristor arrays in simulations.**

The memristor arrays mainly function to implement the weight matrix. An analogue-to-digital converter circuit supplies voltage to the memristorc arrays. The output current is then converted to the voltage signal by a transimpedance amplifier. Then the output voltage is sampled by another analogue-to-digital converter before being transferred to the FPGA.


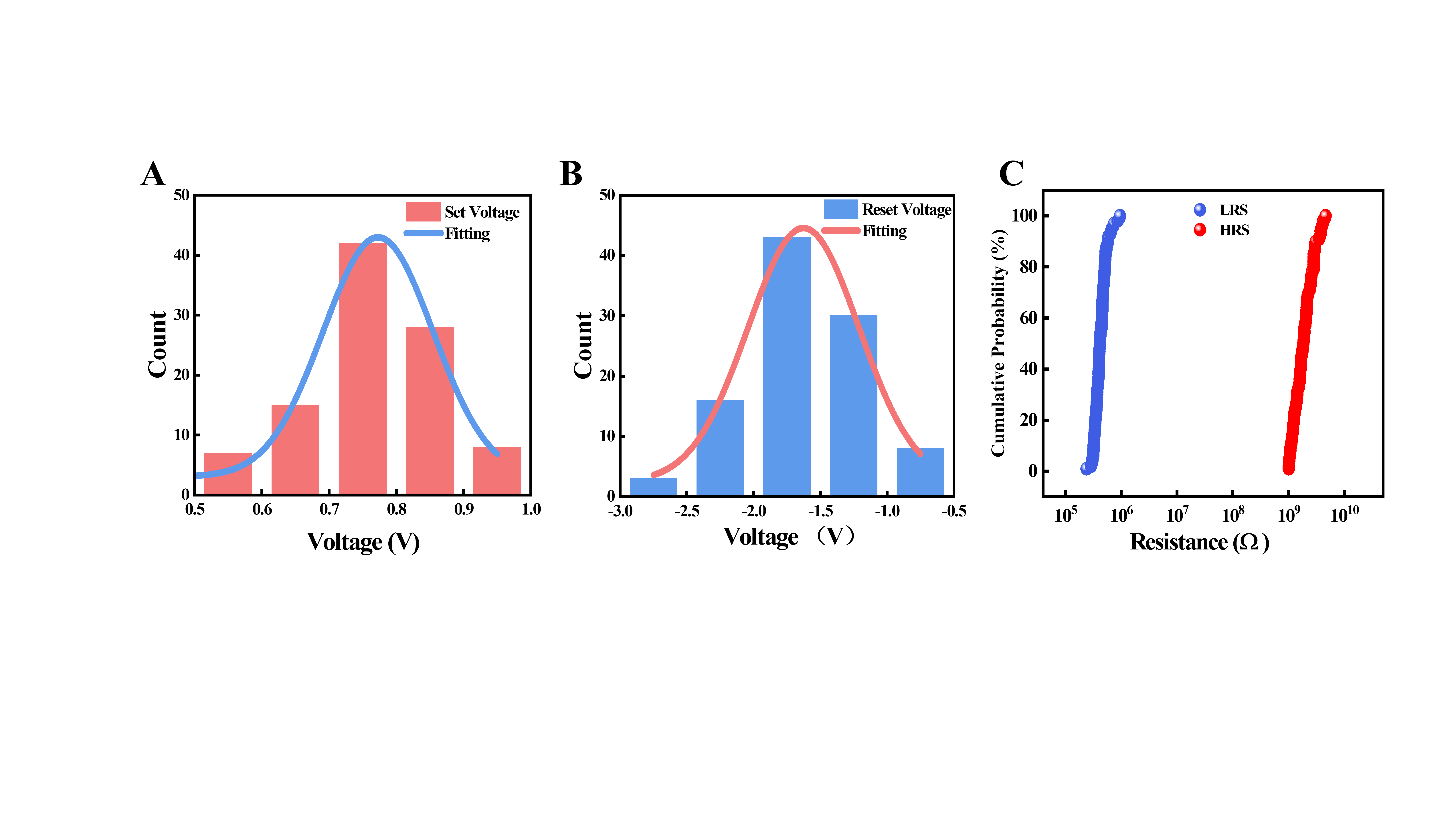


**Fig. S4. The statistical electrical characteristics of the Gd:HfO_2_ based device.** The corresponding histogram of the (A) set and (B) reset voltages fitting by the Normal distribution. (C) The HRS and LRS distributions of the Gd:HfO_2_ based device.

**Table. S1. The NIST test suite results.** As can be seen from the pass rate and P-value, the random number test passed.

| **Name** | **P-value** | **Pass rare** | **Success/failure** |
| --- | --- | --- | --- |
| Block frequency | 0.064392 | 15/16 | Success |
| Cumulative sums | 0.025381 | 15/16 | Success |
| FFT | 0.004075 | 16/16 | Success |
| Frequency | 0.230067 | 16/16 | Success |
| Longest run | 0.175923 | 15/16 | Success |


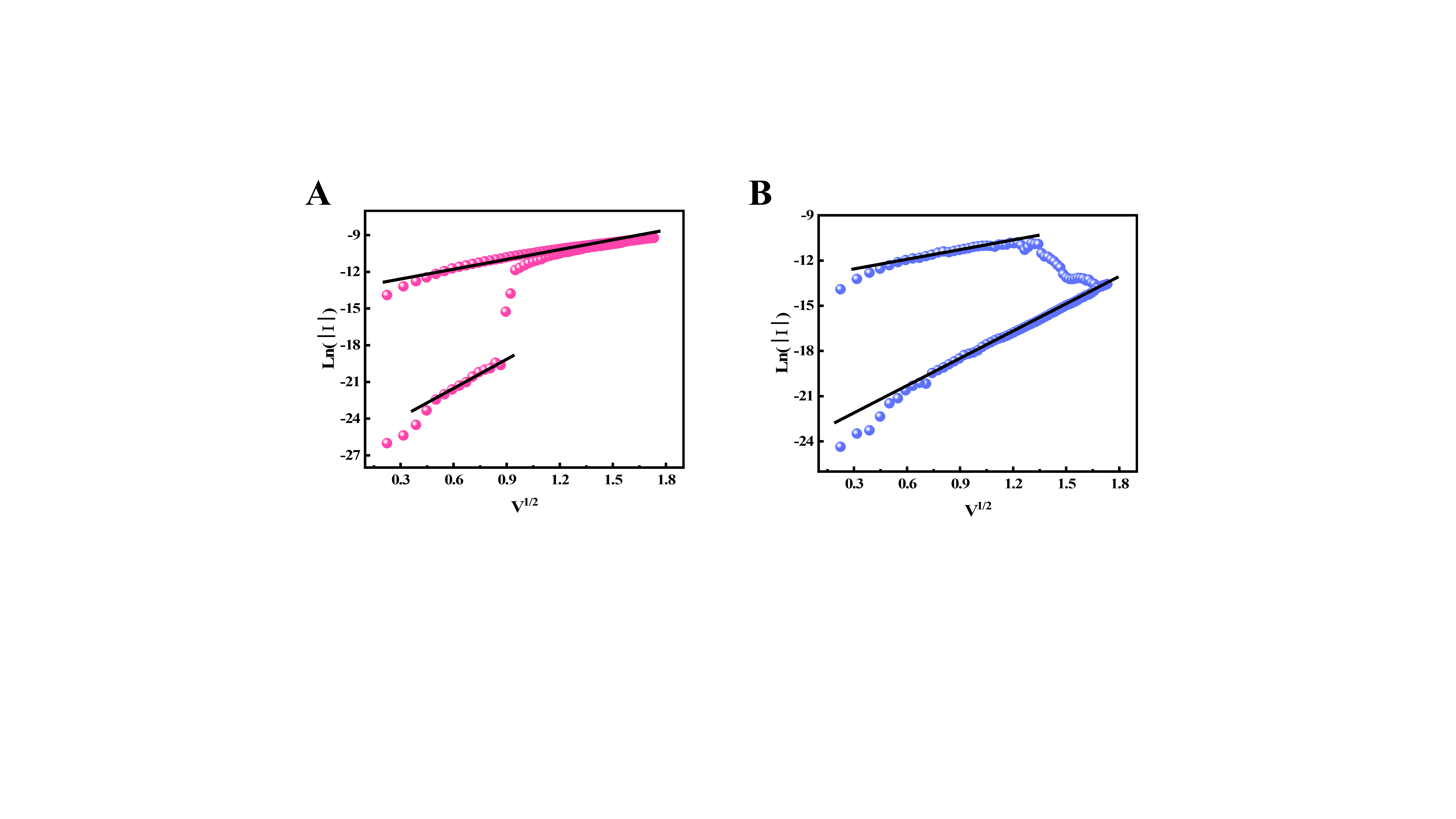


**Fig. S5. The I-V characteristic fitting curve based on the thermionic emission model.** (A) positive voltage scanning. (0→3 →0V). (B) negative voltage scanning. (0→-3 →0V).

The I-V curves were fitted by using the Schottky emission model given below (*1, 2*):

where A is the effective Richardson constant, T is the temperature, q is the electron charge, k is the Boltzmann constant, ε_i_ is the permittivity, φ_B_ is the barrier, d_eff_ is the effective thickness, respectively. The liner relationship of ln (I) versus $\sqrt{V}$was plotted.


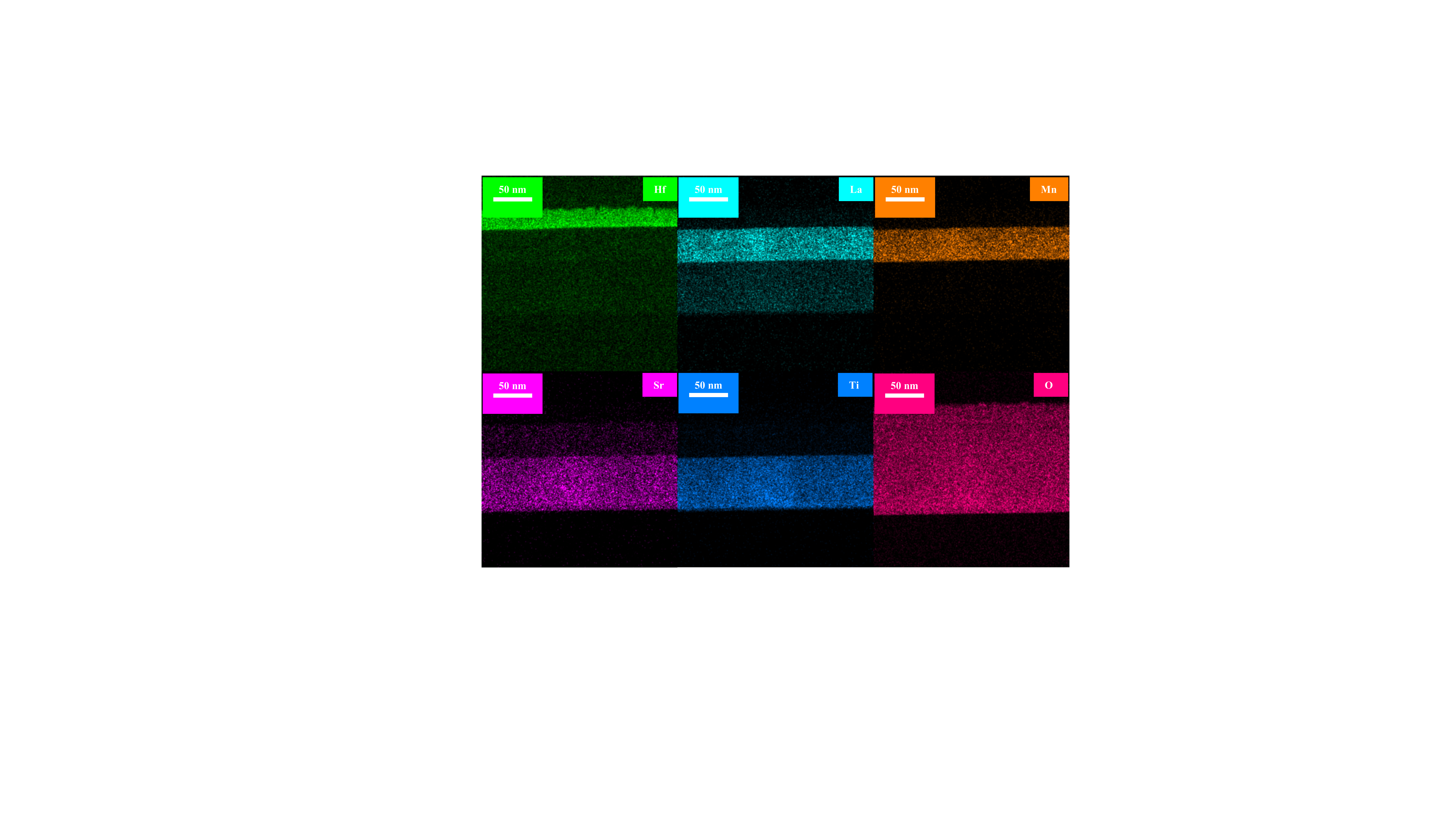


**Fig. S6. The elemental mapping images were used to characterize the element distribution of Gd:HfO_2_ based device.**


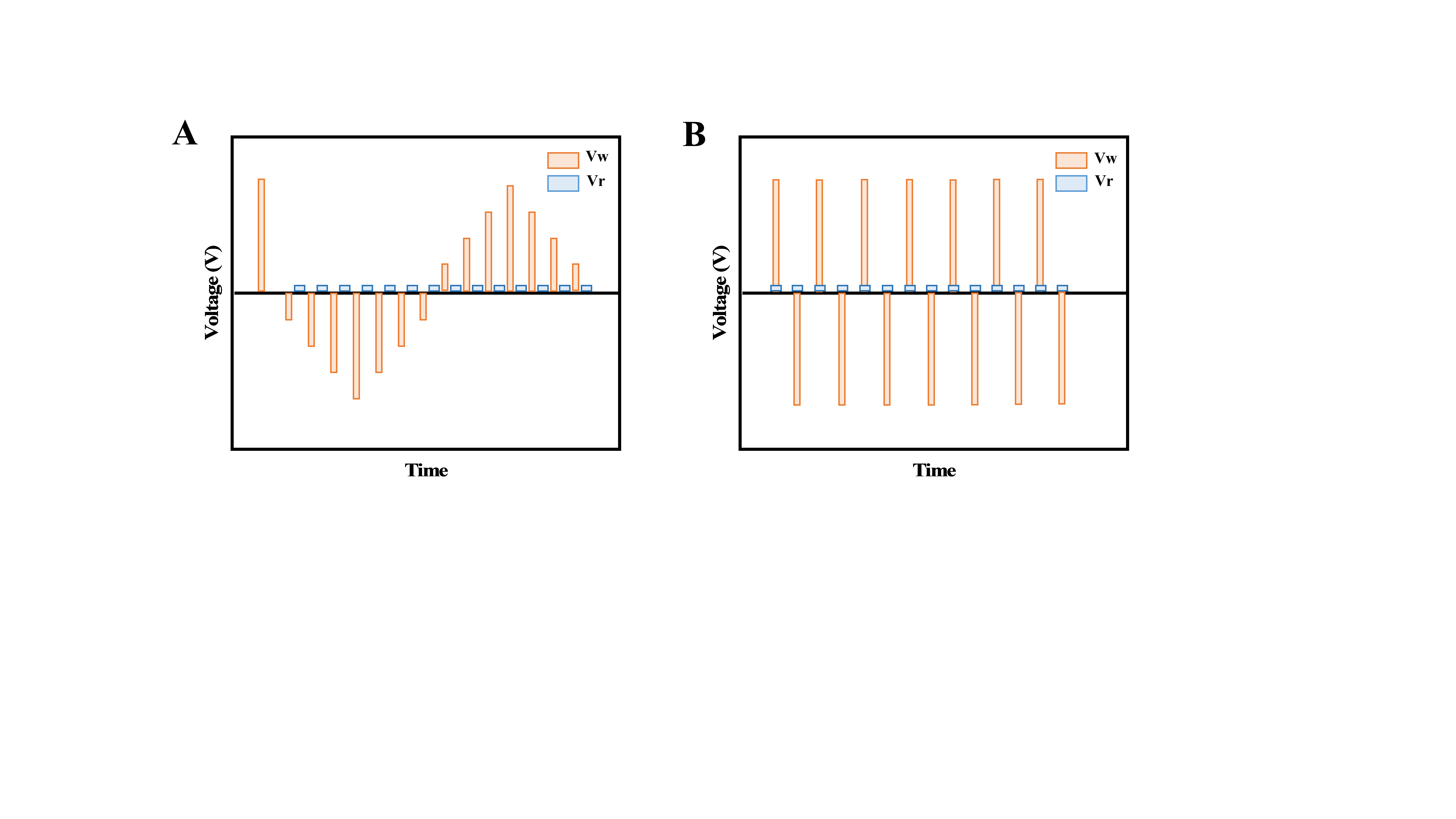


**Fig. S7. The R-V hysteresis loops and cyclability measurements of Gd:HfO_2_ based device.** (A) Applied voltage pulse sequences for the R-V measurement. (B) Applied voltage pulse sequences for the cyclability measurement.


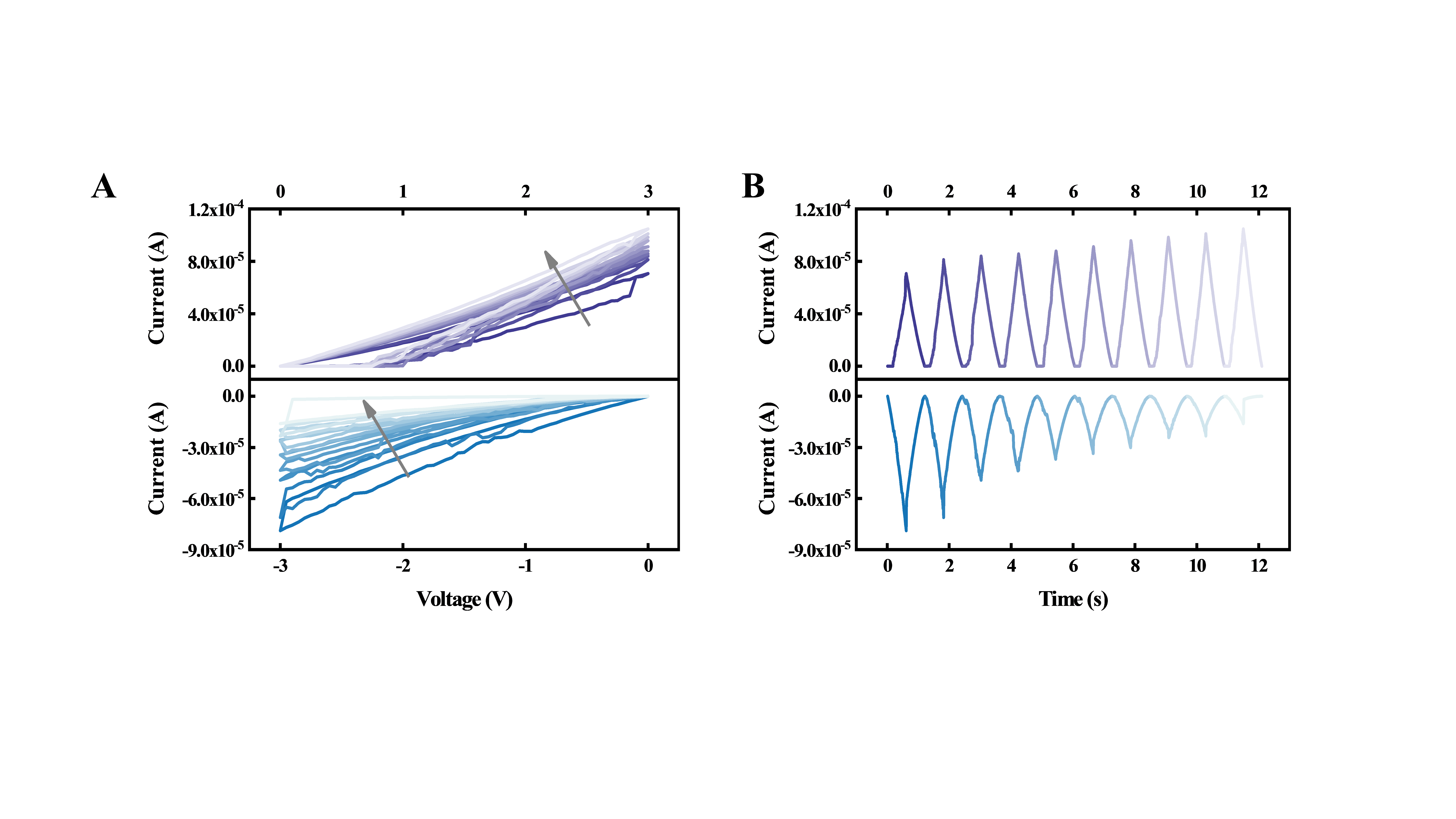


**Fig. S8. The obviously down-up and up-down evolution of conduction as biological synapse is illustrated in positive and negative part of I-V curves.**


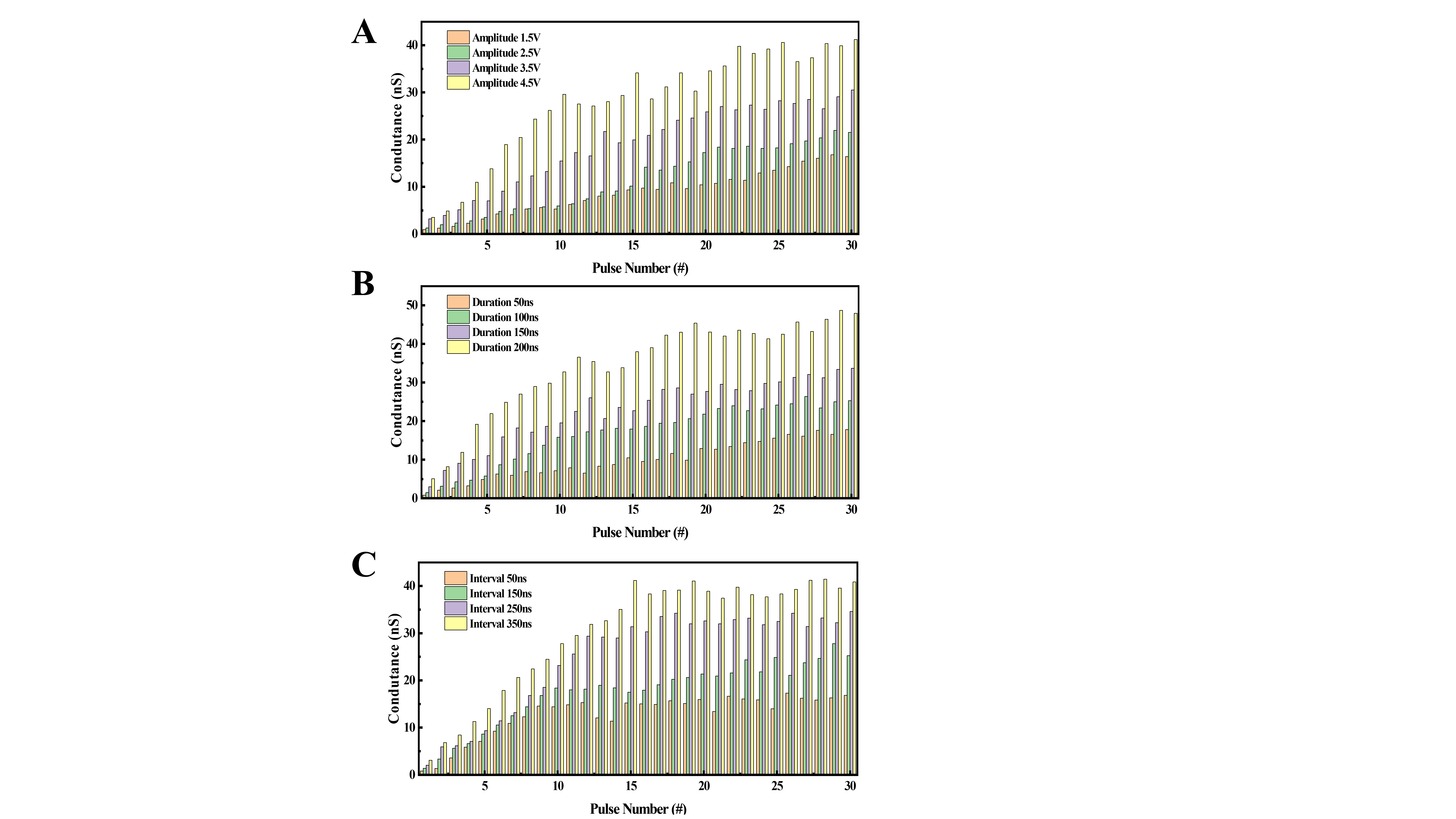


**Fig. S9. The effect of different parameters to adjust the pulse sequence on the conductance of Gd:HfO_2_ based memristor.** The effect of the different pulse (A) amplitude (B) duration (C) interval on the device conductance.


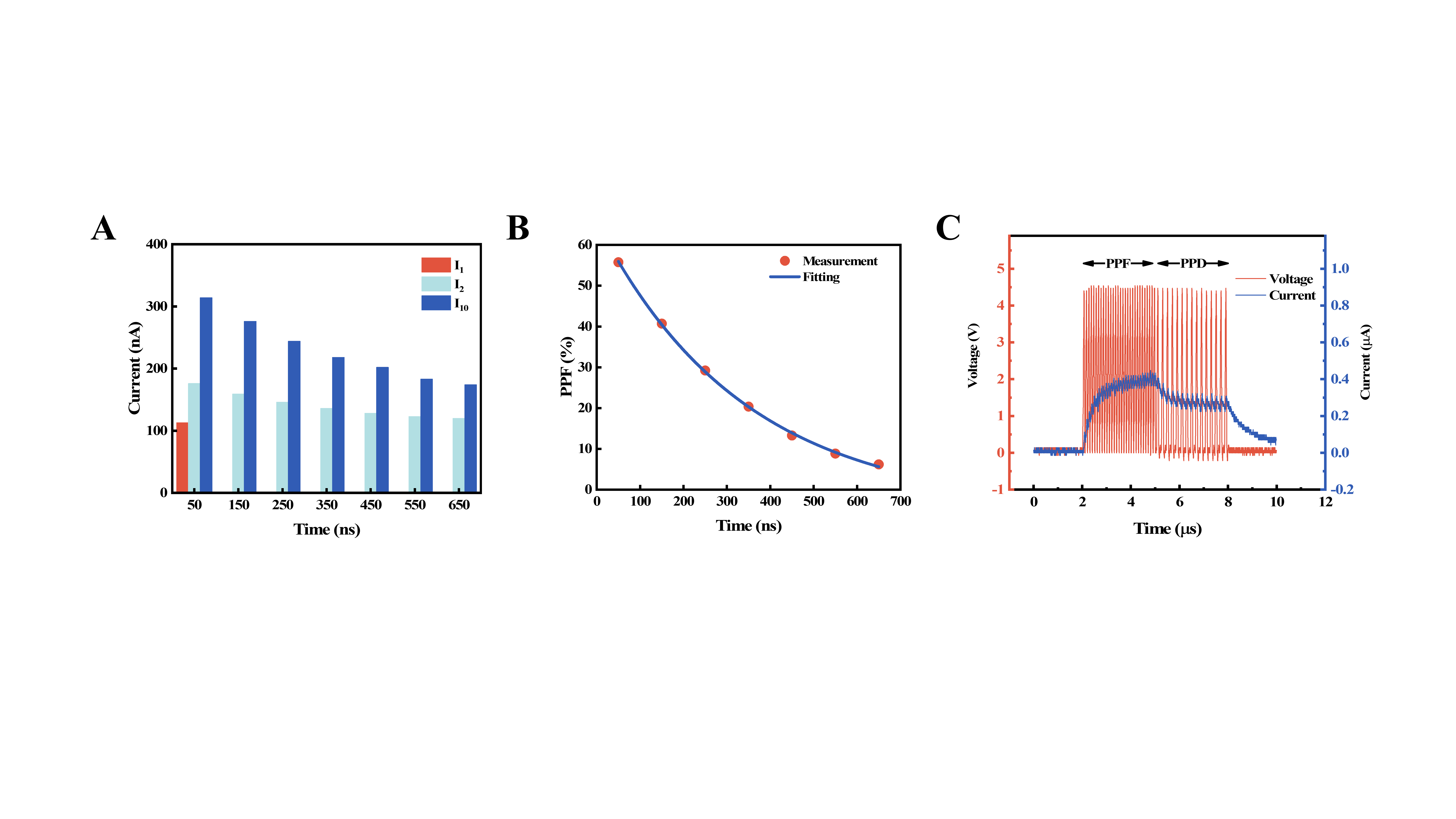


**Fig. S10. The Synaptic functions of Gd:HfO_2_ based device.** (A) The current response at different pulse intervals. (B) The PPF index. (C) The evolution from PPF to PPD by changing the stimulus pulse interval.


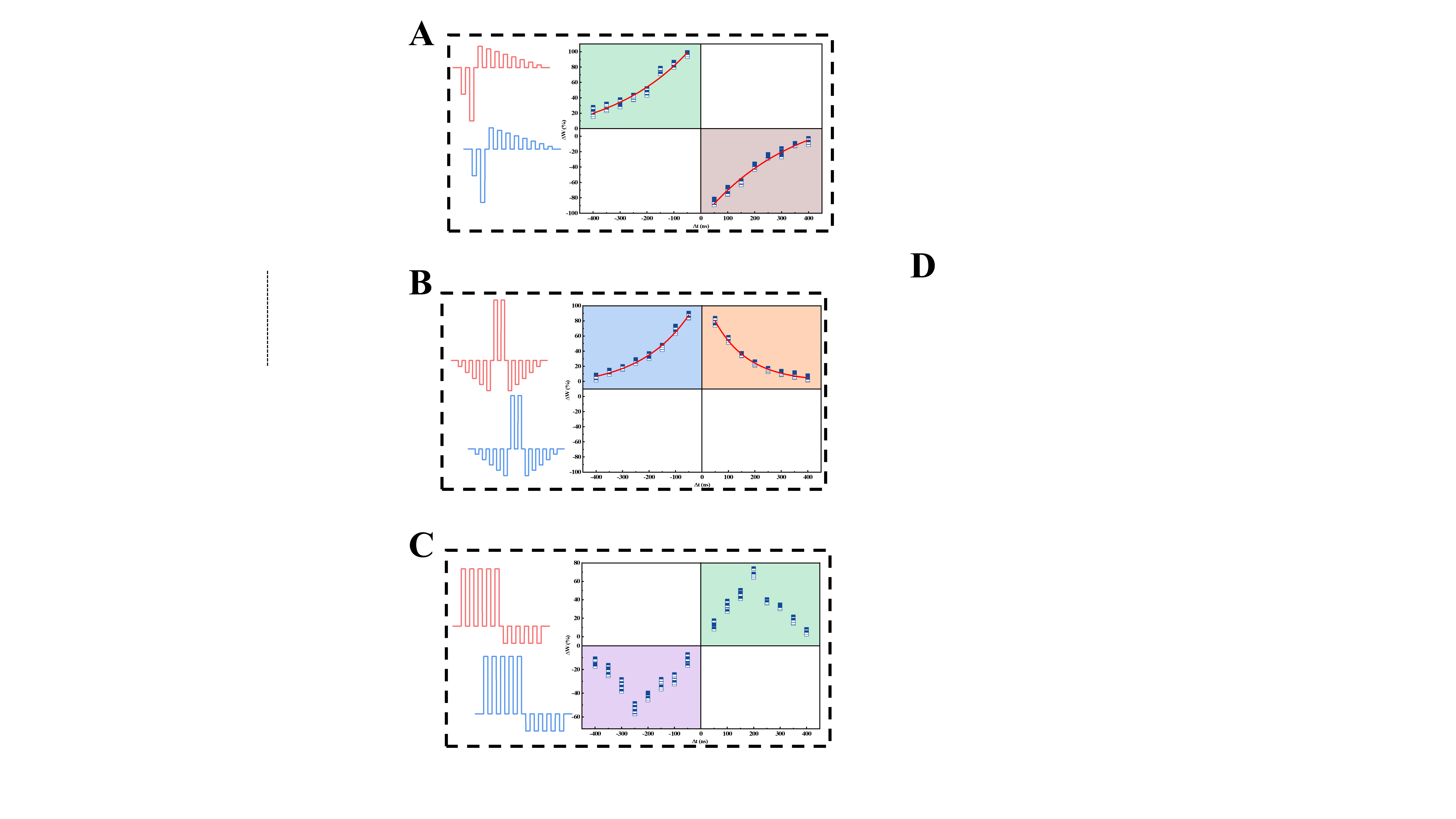


**Fig. S11. The STDP rule measurements.** (A) The Anti-Hebbian STDP pulse train waveform and ΔW as a function of Δt. (B) The Symmetrical STDP pulse train waveform and ΔW as a function of Δt. (C) The Visual STDP pulse train waveform and ΔW as a function of Δt.


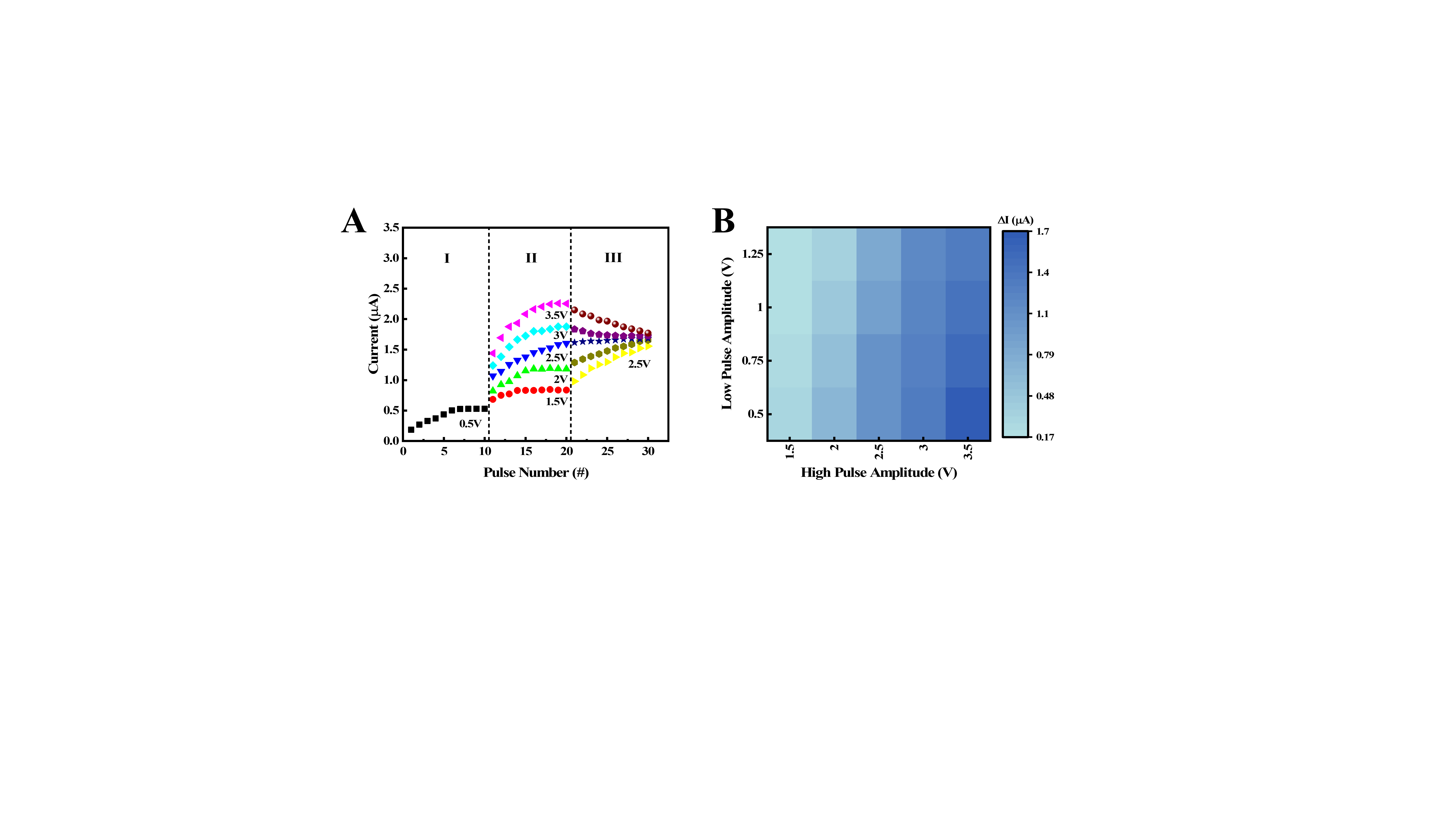


**Fig. S12. The SADP functional simulation.** (A) The synaptic weight response to a set of spike trains with an amplitude sequence starting from 0.5 V. (B) The change of synaptic weight under different high pulse amplitudes and low pulse amplitudes.


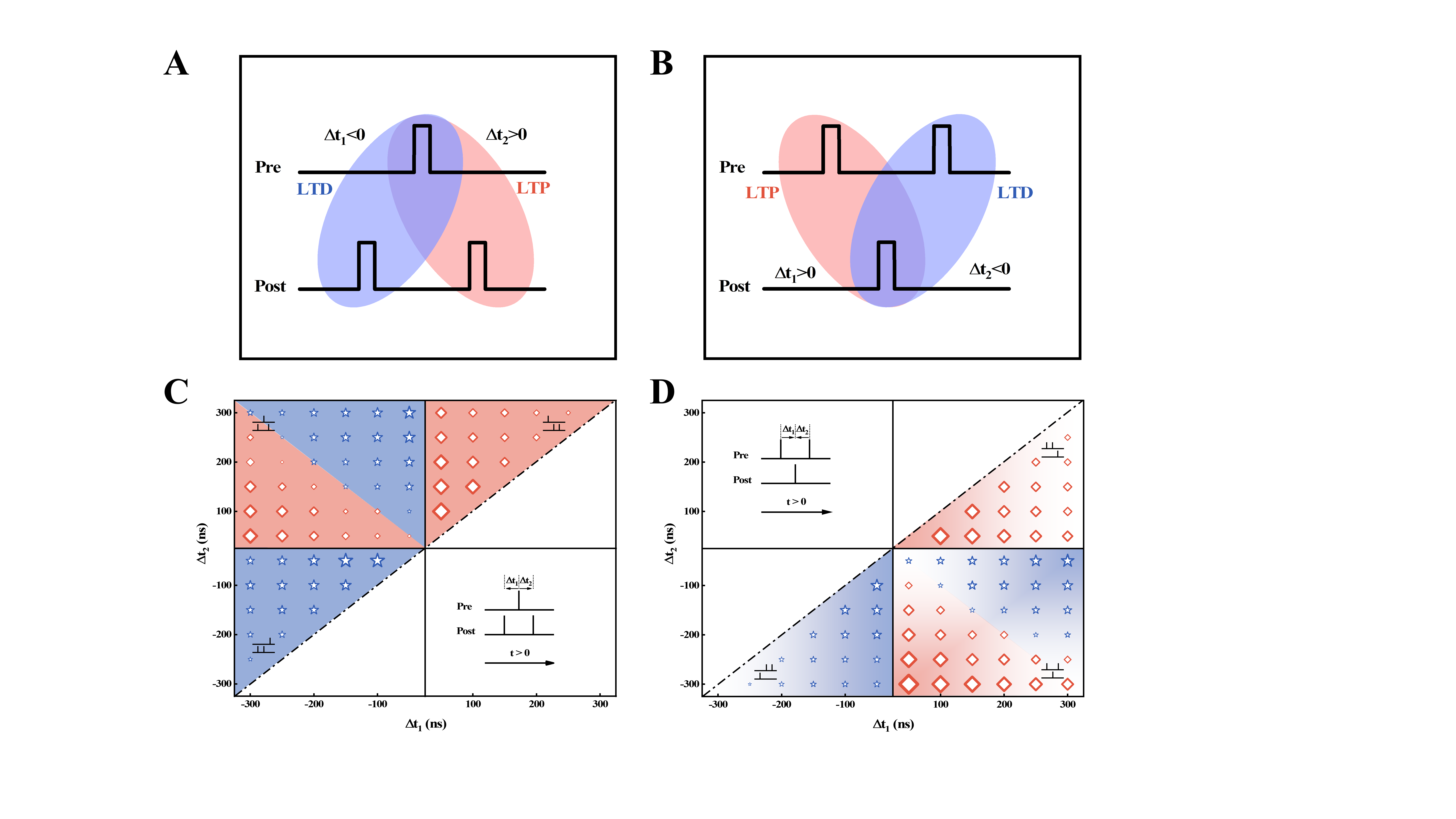


**Fig. S13. The BCM learning rule could be achieved by the triplet-STDP model.** The diagram of the (A) “post−pre−post” and (B) “pre−post−pre” triplets. (C) (D) The triplet-STDP results with different pulse intervals and pulse sequences.


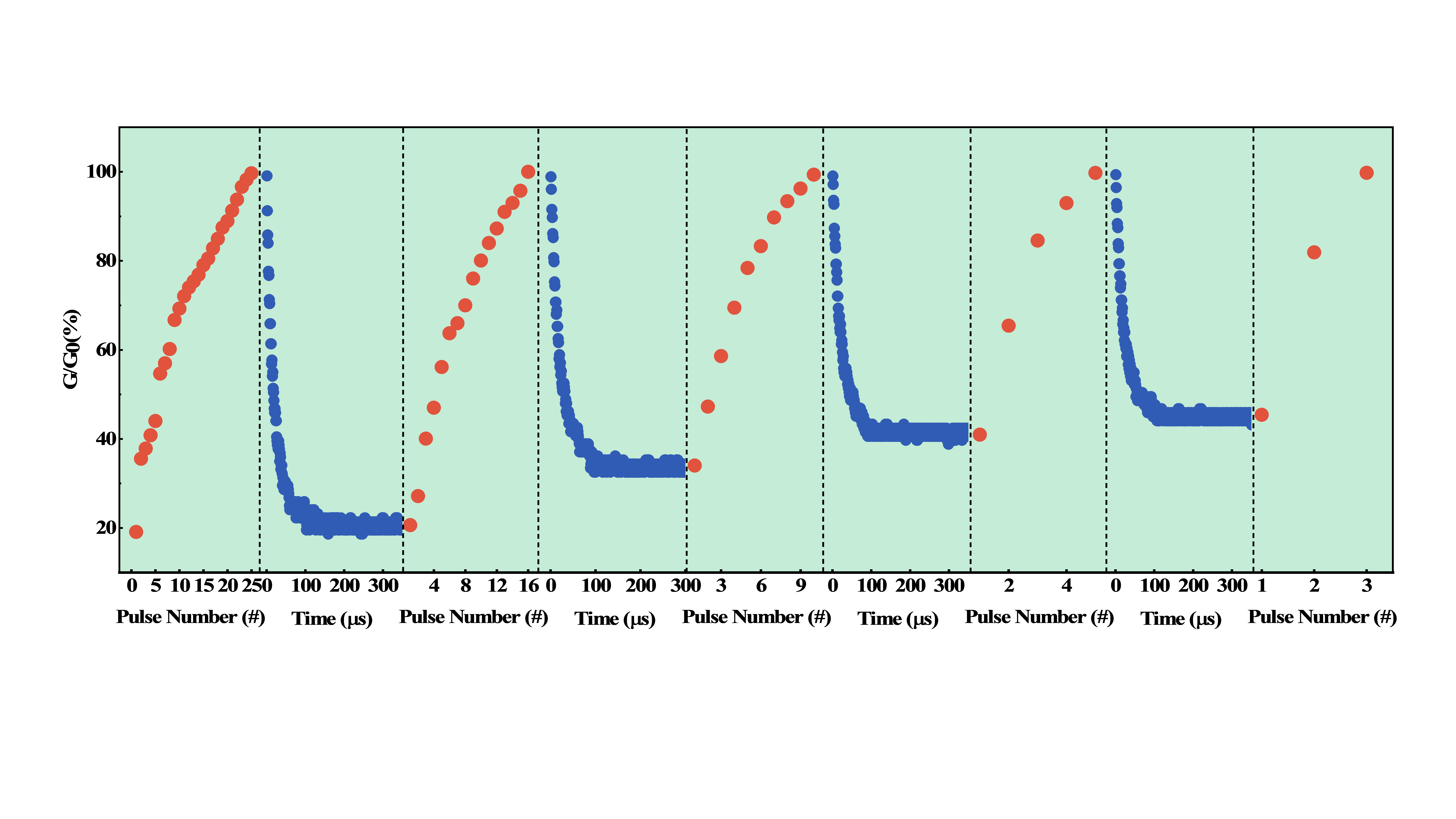


**Fig. S14. The simulation of learning-forgetting-relearning characteristics in the human brain.**

**Table. S2. Comparison of the parameters with other memristor devices.**

| Device structure | Set/Reset  voltage | Power  consumption | Switching speed | Retention time | Endurance | multi-value storage | Ref. |
| --- | --- | --- | --- | --- | --- | --- | --- |
| Au/HZO/Si | 1.8 V/-1.8 V | - | - | 7.2×10^3^ s | 10^3^ | 5 | (*3*) |
| ITO/α-IGZO/  Al:HfO_2_/W | - |  | ~100 ns | 10^4^ s | - | 2 | (*4*) |
| TiN/HZO/Pt | - | 1.8pJ | ~100 ns | 10^4^ s | - | 2 | (*5*) |
| TiN/HZO/Al_2_O_3_/TiN | 3 V/-2 V | - | - | 10^4^ s | - | 4 | (*6*) |
| p^+^-Si/HZO/TiN | - | - | - | - | 10^4^ | 2 | (*7*) |
| Al/IGZO/HZO/TiN | - | 1.5 nJ | - | 10^4^ s | - | - | (*8*) |
| TiN/HZO/TiN | - | 12.5 pJ | - | 4×10^3^ s | - | 2 | *(9)* |
| Pt/Al_2_O_3_/HfO_2_/  HfAlO_x_/TiN | -3 V/2.4 V | - | - | 10^4^ s | 10^3^ | 7 | (*10*) |
| n^++^ Si/TiN/HZO/  GeSn/Ni | - | - | - | 10^8^ s | 10^6^ | 2 | (*11*) |
| TiN/HZO/Pt | -1.7 V/+2.2V | - | - | >3×10^4^ s | 10^3^ | 2 | (*12*) |
| LSMO/BTO/LSMO | 2.2 V/-2 V | 35 pJ | 6 ns | - | - | 5 | (*13*) |
| Au/Ti/ZnO/BFO/  SRO/BTO/mica | 2 V/-2 V | - | - | 10^4^ s | - | 4 | (*14*) |
| Au/P(VDF-TrFE)/  Al_2_O_3_/n^++^ Si | - | 1.2 pJ | 1.1 ms | < 10^3^ s | - | 2 | (*15*) |
| Au/Pt/BFO/SRO | 6.3 V/-2.3 V | - | ~100 ns | 10^5^ s | 10^5^ | 2 | (*16*) |
| Au/SnSe/NSTO | - | 66 fJ | - | >10^4^ s | - | 2 | (*17*) |
| Ti/Au/α-In_2_Se_3_/  Ti/Au | - | ~0.8 nJ | - | 2×10^5^ s | 10^3^ | 2 | (*18*) |
| Pd/Gd:HfO_2_/LSMO/  STO/Si | 0.75 V/-1.75 V | 2.07 fJ | 20 ns | 10^4^ s | 10^9^ | 16 | This work |


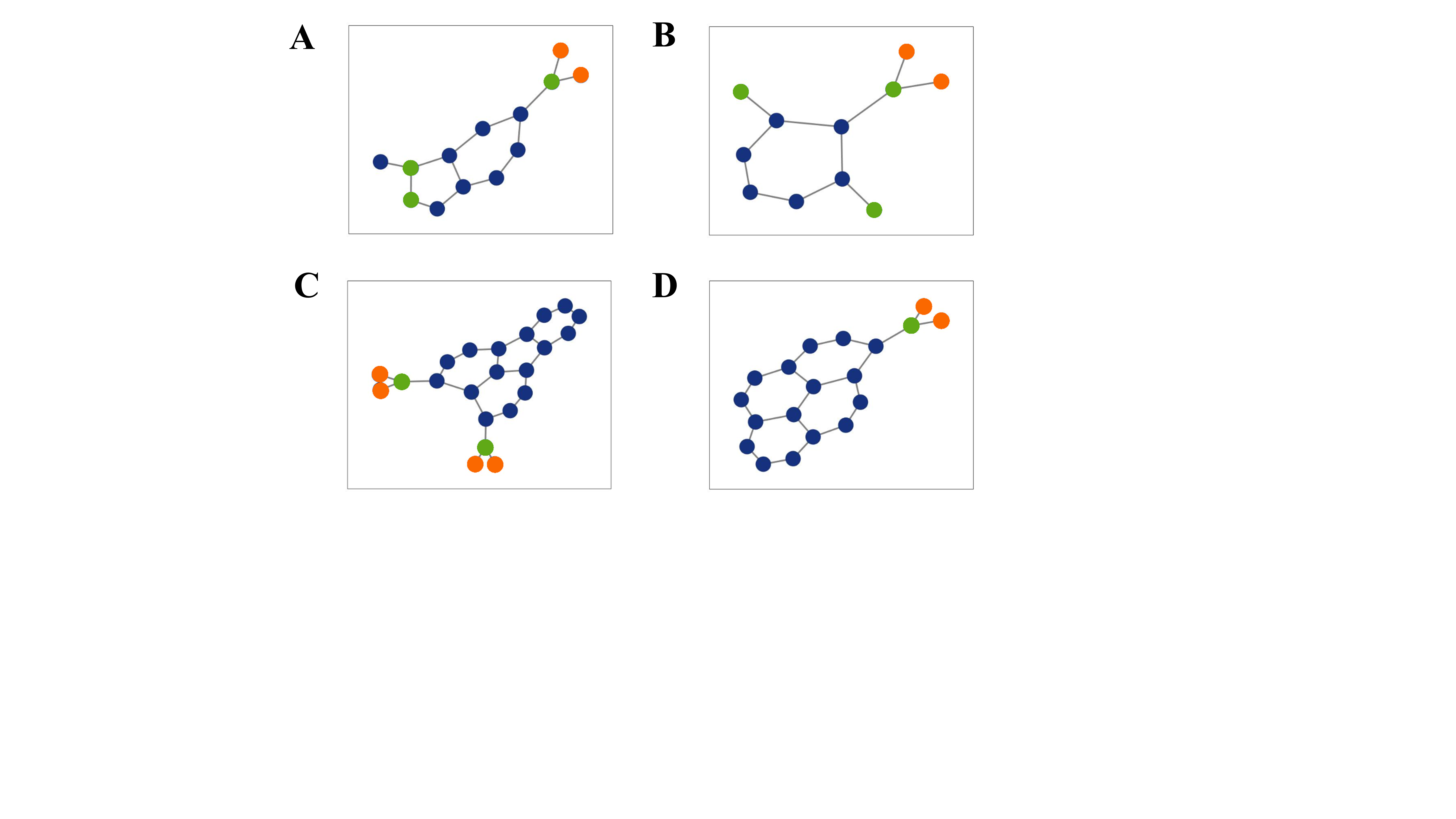


**Fig. S15. The schematic diagram of the molecular structures in MUTAG dataset.**

Unlike image, graph-structured data consists of nodes and edges. The MUTAG dataset contains 188 graph-structured data, each of which represents a nitro compound molecule. The nodes represent atoms and different colors represent different elements. There are seven atom types, the input features of each node are represented by 7-dimensional vectors, with edges representing chemical bonds by using adjacency matrix.

Given a graph$G=(V,E)$, where$V=\{v_{1},v_{2},...,v_{N}\}$is the set of nodes,$E=\{(u,v)\left|u\in V,v\in V\right.\}$is the set of edges in $G$. The adjacent matrix is $N\times N$ square matrix, defined as:

$$A_{i,j}=\left\{\begin{matrix} 1,\text{(}v_{i},v_{j}\text{)}\in E \\ 0,\text{(}v_{i},v_{j}\text{)}\notin E \end{matrix}\right.$$

The feature representation of graph structured sample is made up of two arrays: node array (storing vertices information of the graph) and adjacent matrix (storing edges or arcs information of the graph).


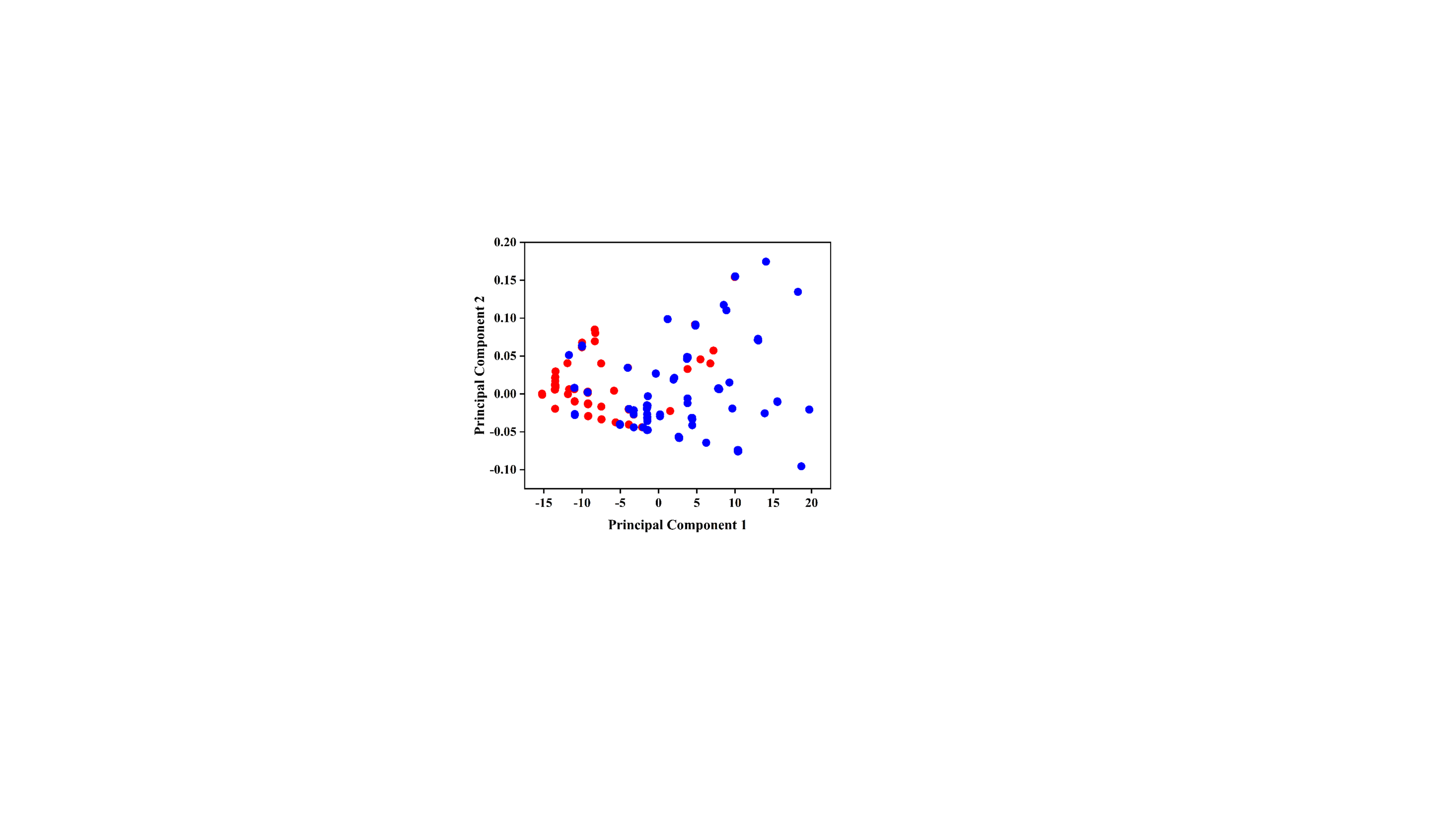


**Fig. S16. The 2D visualization of the graph feature representation in the MUTAG dataset.**

The 60-dimensional graph feature representation was mapped to a 2-dimensional space using PCA. Blue color indicates positive classes and red color indicates negative classes. Points of the same color are clustered together so that points of different colors can be linearly separable.


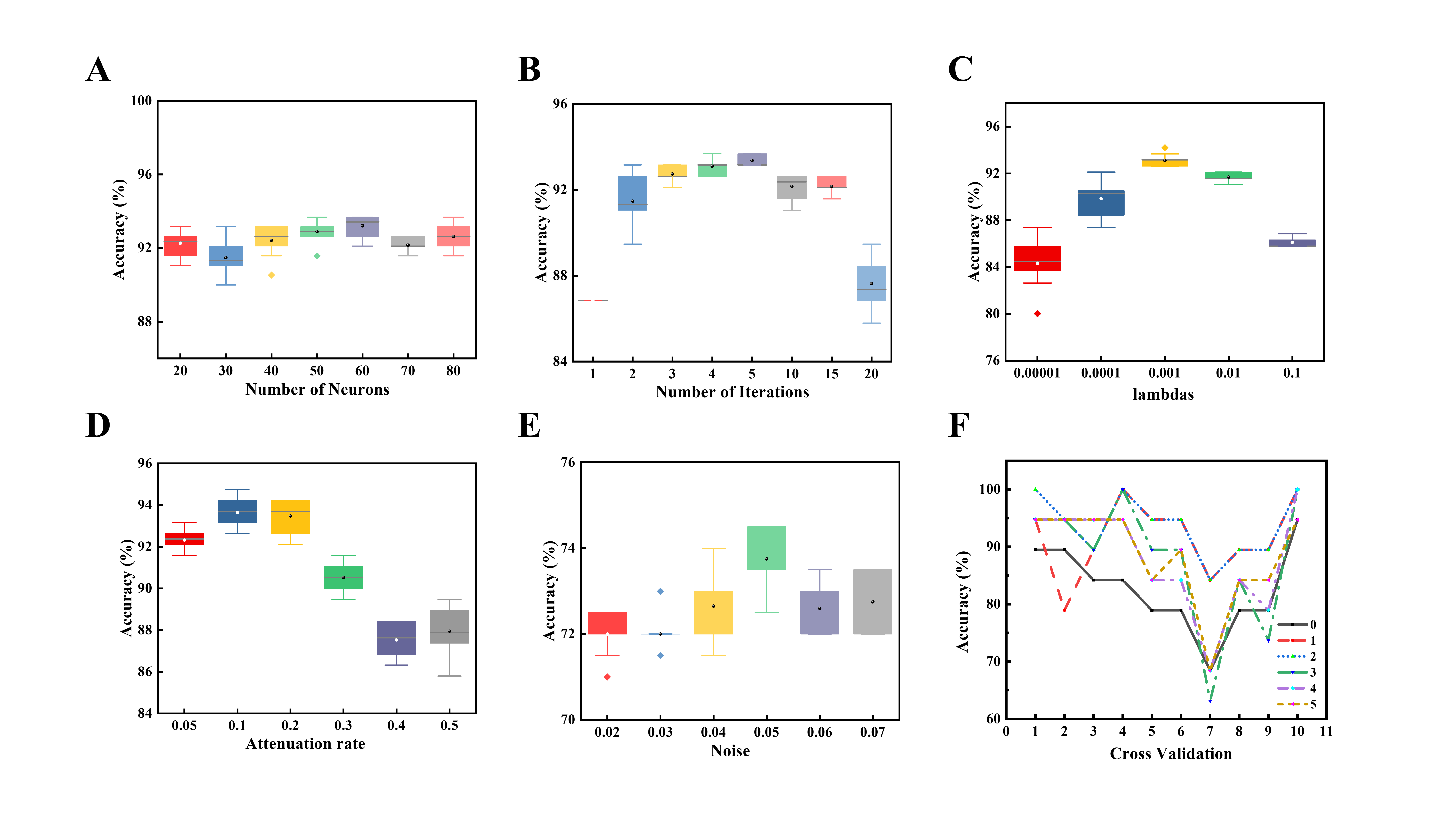


**Fig. S17. The impact of hyperparameters on the performance of MUTAG classification task.** (A) The effect of the number of neurons in the echo layer on performance. The network performs best when the number of neurons is 60. (B) The effect of the number of iterations in the echo layer on performance. The performance is best when the number of iterations is 5. Too few iterations do not adequately learn the topological information of the graph data. Too many iterations create over-smoothing problems. (C) The effect of the tuning parameter of the regularization term in the readout layer on the performance. The tuning parameter controls the degree of network fitting and the degree of contraction of the regression coefficients, and the best performance is achieved when the tuning parameter is 0.001. (D) The effect of the leakage coefficient in the forward propagation process on the performance, with the best performance at 0.1. (E) The effect of noise size on performance during initialization of network weights. The best performance is achieved by increasing the noise size to 0.05. (F) The effect of different trade-off parameters in the forward propagation process on the network performance in ten cross-validations, and the best performance is achieved when the trade-off parameter is 2.


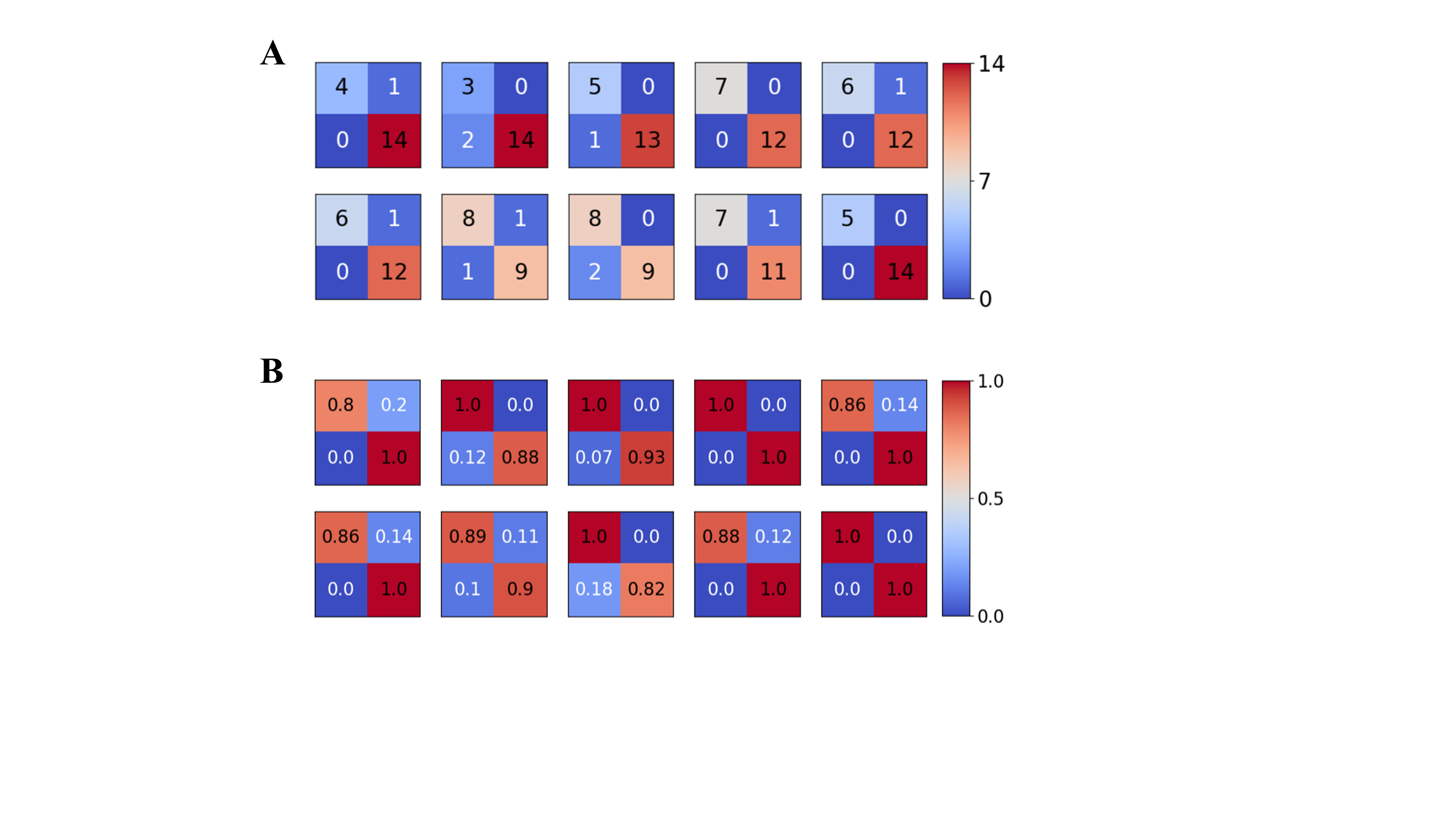


**Fig. S18. The confusion matrix for MUTAG classification results in the 10-fold cross-validation. The rows represent true categories and columns represent predicted categories.** (A) Fold-wise confusion matrices in classifying the MUTAG dataset. The higher the value of the diagonal line, the higher the classification accuracy. (B) The normalized fold-wise confusion matrices in classifying the MUTAG dataset. Most of the graph-structured data can be classified accurately.


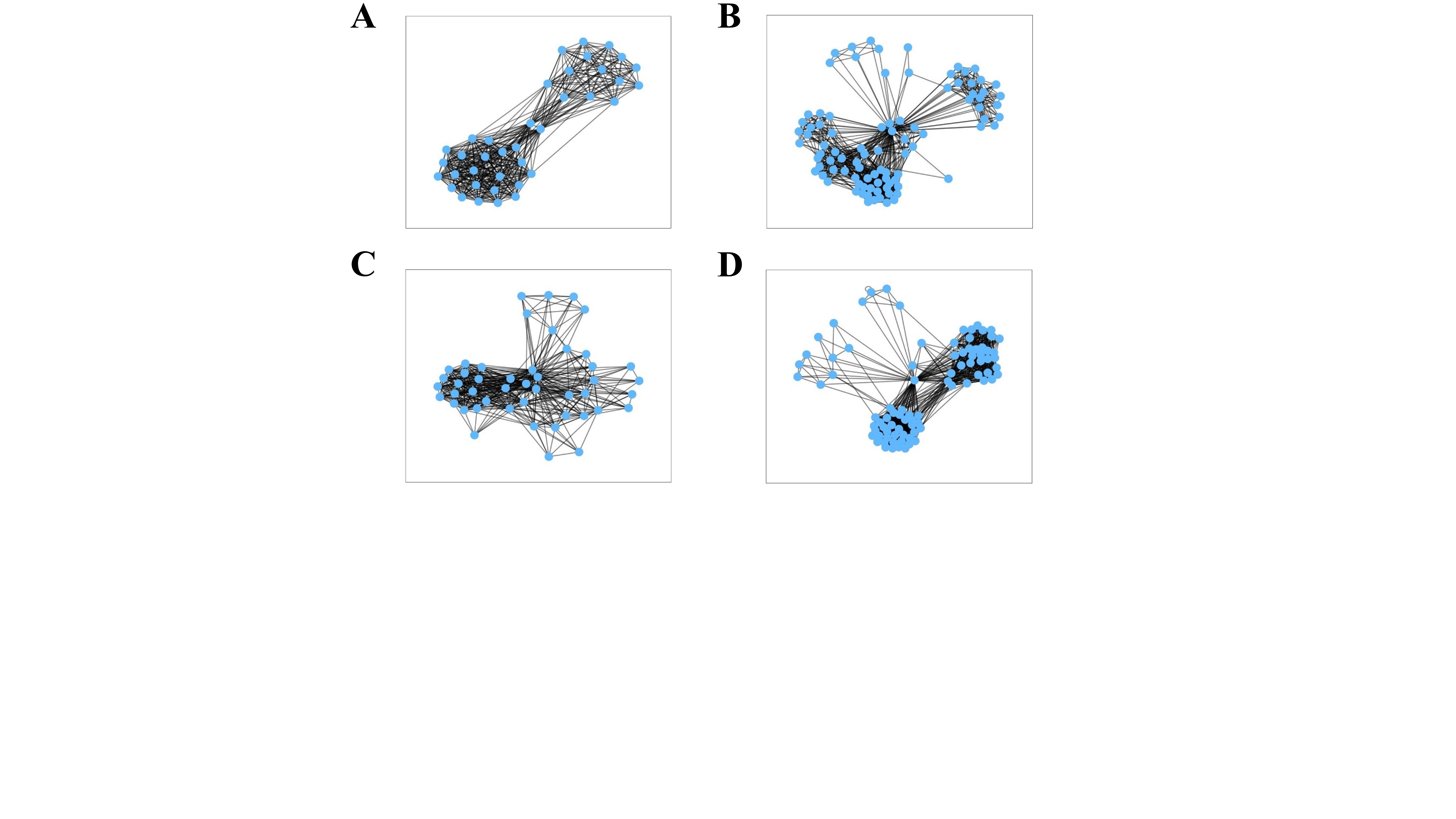


**Fig. S19. The schematic diagram of the social network structures in COLLAB dataset.**

The COLLAB dataset contains 5000 graph-structured data, where each graph corresponds to a researcher’s ego network. The nodes represent the researchers and its collaborators. The input features of each node are represented by 1-dimensional vectors. The edges represent collaborations between two researchers, and the edges are represented by adjacency matrices. The dataset is categorized as high energy physics, condensed matter physics, or astrophysics, depending on the field to which the researcher belongs.


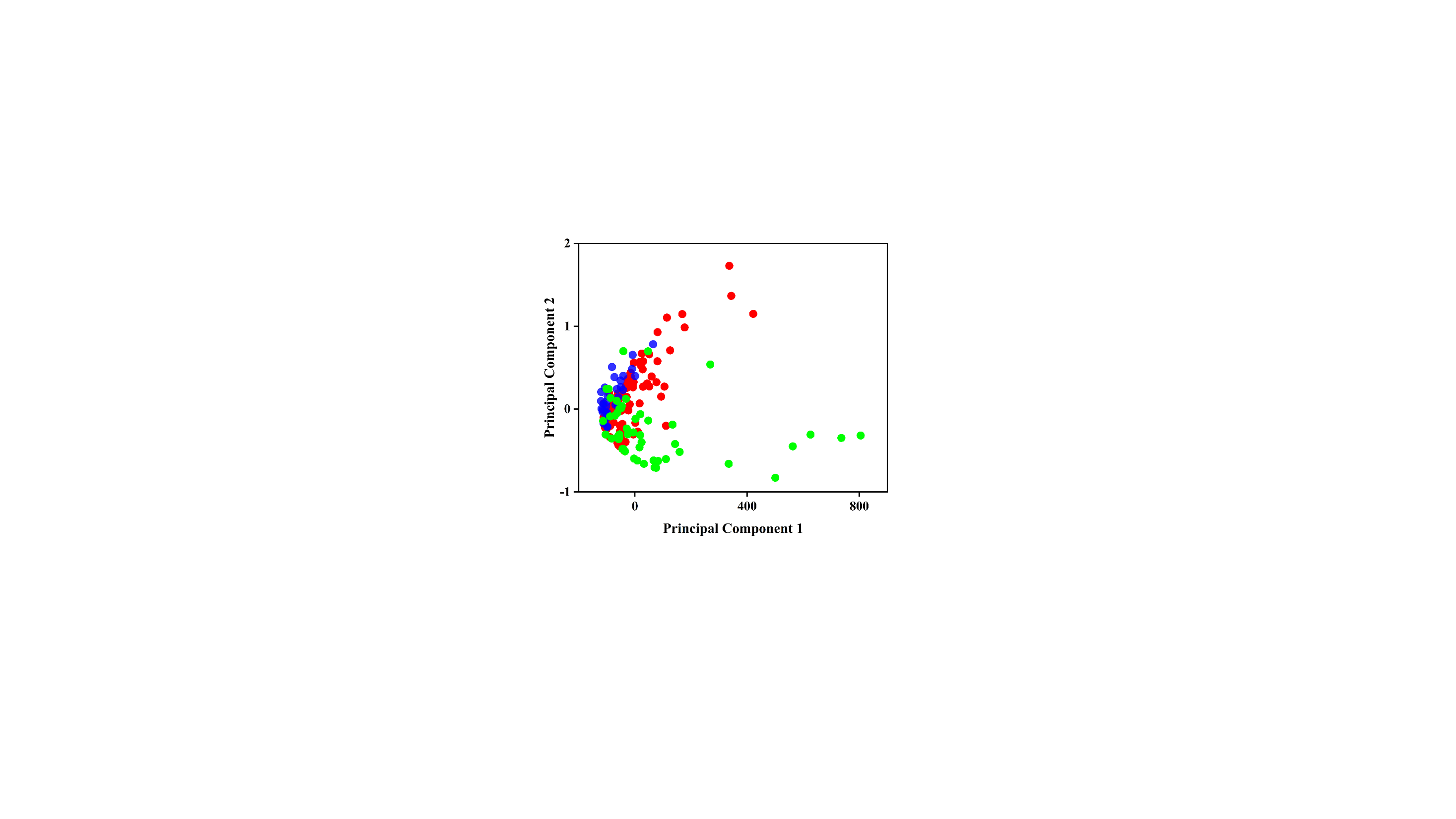


**Fig. S20. The 2D visualization of the graph feature representation in the COLLAB dataset.**

For the COLLAB dataset, the graph-structured data possesses a 70-dimensional feature representation. The graph feature representation was downscaled to 2-dimensional space using PCA. The red points is astrophysics, the blue points is condensed matter physics, and the green points is high energy physics. Most of the green and blue points are linearly separable, and the red points partially overlap with points of the other two colors.


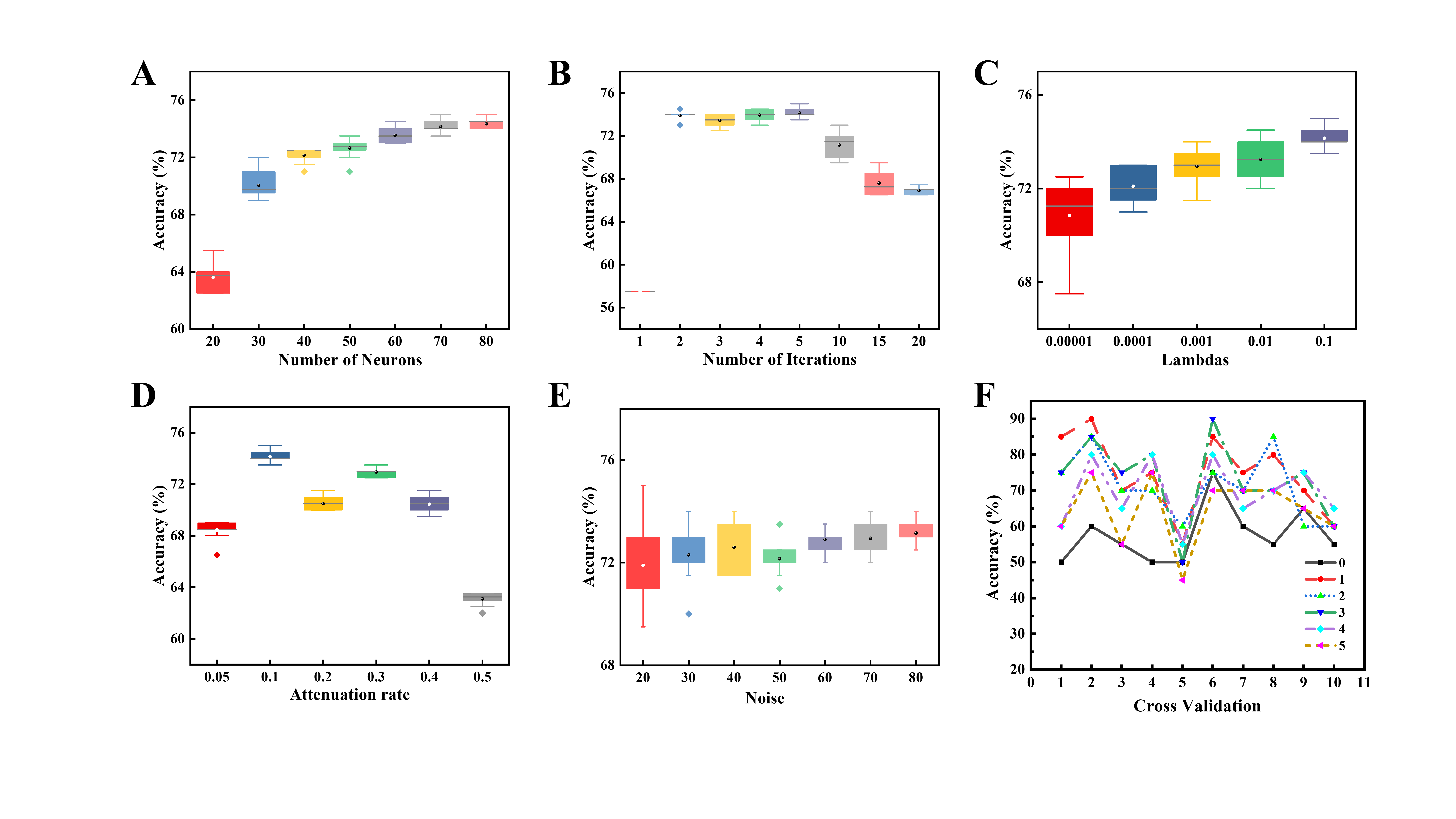


**Fig. S21. The impact of hyperparameters on performance of the COLLAB classification task.** (A) The effect of the number of neurons in the echo layer on performance. The network performs best when the number of neurons is 70 for classification on the COLLAB dataset. (B) The effect of the number of iterations in the echo layer on performance. The optimal number of iterations is the same as the MUTAG dataset for the classification task, and the best performance is achieved when the number of iterations is 5. (C) The effect of the tuning parameter of the regularization term in the readout layer on the performance. The best performance is achieved when the tuning parameter is 0.1. (D) The effect of leakage coefficient on performance during forward propagation, which is the same as that of the MUTAG dataset for the classification task, and the best performance is achieved when the leakage coefficient is 0.1. (E) The effect of noise size on performance during initialization of network weights. Increasing the noise size to 0.05 gives the best performance. The effect of adding different noise does not change much on the network performance. (F) The effect of different trade-off parameters during forward propagation on network performance in ten cross validations, with the best performance when the trade-off parameter is 3.


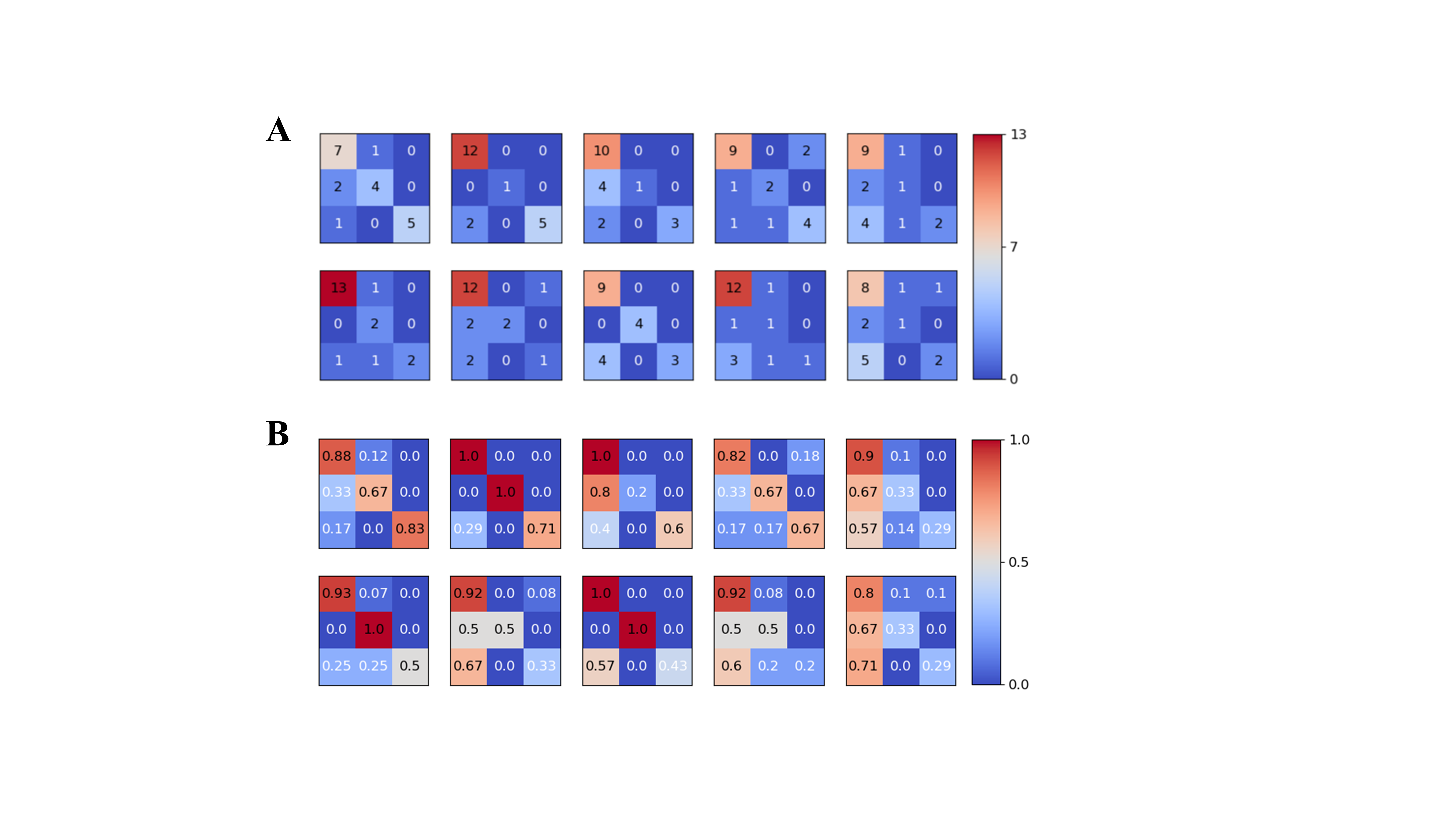


**Fig. S22. The confusion matrix for COLLAB classification results in the 10-fold cross-validation. The rows represent astrophysics, condensed matter physics, and high energy physics, respectively. The columns represent predicted categories.** (A) Fold-wise confusion matrices in classifying the COLLAB dataset. In each fold-wise confusion matrix, astrophysics is able to be accurately classified, while condensed matter physics and high energy physics are often misclassified as astrophysics. (B) Normalized fold-wise confusion matrices in classifying the COLLAB dataset.


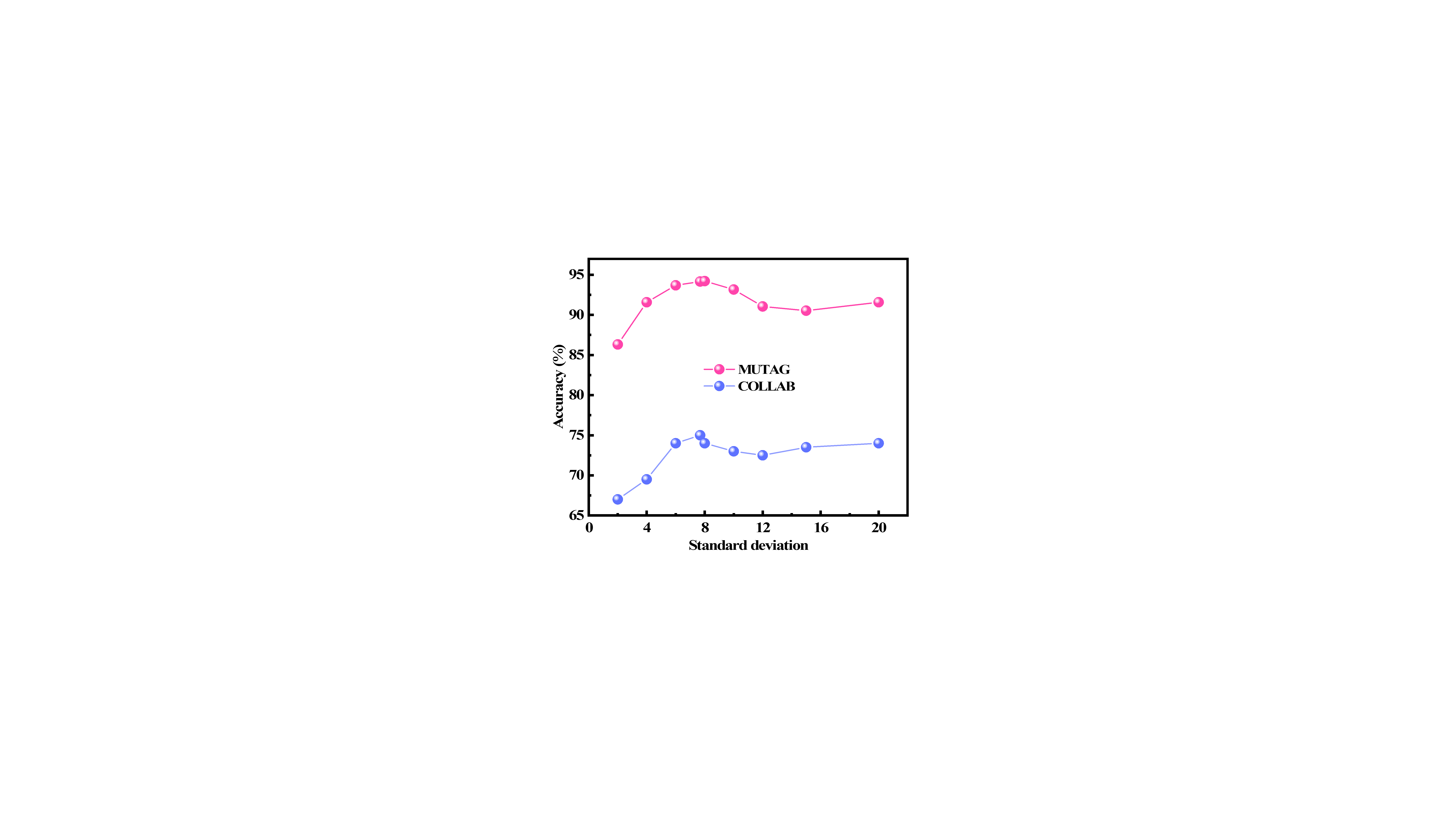


**Fig. S23. The effect of standard deviation on accuracy.** The experimentally measured conductance conforms to a Gaussian distribution, which tends to have both large and small degrees of dispersion, neither of which is conducive to classification accuracy. The conductance of our Gd:HfO_2_ memristors has suitable degree of dispersion (standard deviation = 7.68 nS), obeying a suitable Gaussian distribution, and is capable of obtaining optimal classification accuracy.


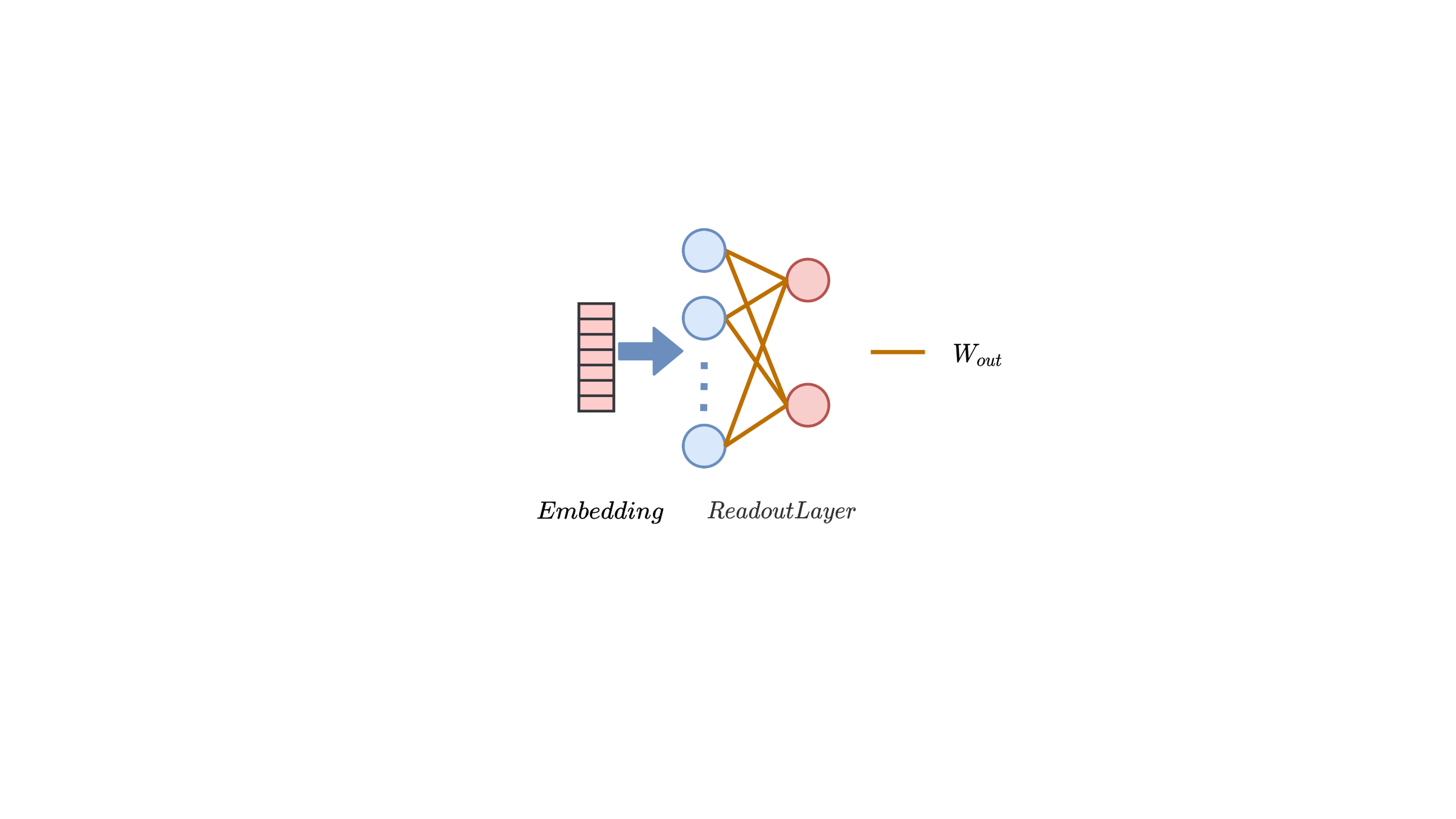


**Fig. S24. The schematic diagram of readout layer.**

The graph-structured data is passed through the echo layer to obtain a graph feature representation. The graph feature representation is passed through the readout layer to obtain the predicted classification results. The readout layer is a fully connected layer, where the number of inputting neurons is the dimension of the graph feature representation and the number of outputting neurons is the number of categories in the dataset. The optimal network connection weights for the readout layer are calculated using ridge regression which is the least squares with L2 regularization. Ridge regression obtains the optimal network connection weights by minimizing the distance between the predicted labels and the corresponding true labels of the graph data. The formula is expressed as follows:

$$\left\|y-w_{out}\mathbf{x}\right\|^{2}-\lambda\left\|w_{out}\right\|^{2}$$

$y$is the real label of the graph-structured data, $w_{out}$is the network connection weight of the readout layer, $\mathbf{x}$is the feature representation of the graph-structured data, and $\left\|w_{out}\right\|^{2}$ is a regularization term to prevent the network from overfitting. Derivation of the above equation is performed to obtain the optimal solution. The specific derivation process is as follows:

$$\begin{matrix} \frac{\left\|y-w_{out}x\right\|^{2}-\lambda\left\|w_{out}\right\|^{2}}{\partial w_{out}}=0 \\ \frac{(y-w_{out}x)^{T}(y-w_{out}x)-\lambda w_{out}^{T}w_{out}}{\partial w_{out}}=0 \\ \frac{y^{T}y-x^{T}w_{out}^{T}y-y^{T}w_{out}x+x^{T}w_{out}^{T}w_{out}x-\lambda w_{out}^{T}w_{out}}{\partial w_{out}}=0 \\ -yx^{T}-yx^{T}+(w_{out}xx^{T}+w_{out}xx^{T})-2\lambda w_{out}=0 \\ -2yx^{T}+2w_{out}xx^{T}-2\lambda w_{out}=0 \\ w_{out}(xx^{T}-\lambda I)=yx^{T} \\ w_{out}=yx^{T}(xx^{T}-\lambda I)^{-1} \end{matrix}$$

References

1. D. Pantel, M. Alexe, Electroresistance effects in ferroelectric tunnel barriers. *Physical Review B* **82**, 134105 (2010).

2. Z. Peng, F. Wu, L. Jiang, G. Cao, B. Jiang, G. Cheng, S. Ke, K. C. Chang, L. Li, C. Ye, HfO2‐Based Memristor as an Artificial Synapse for Neuromorphic Computing with Tri‐Layer HfO2/BiFeO3/HfO2 Design. *Advanced Functional Materials* **31**, 2107131 (2021).

3. T. Yu, F. He, J. Zhao, Z. Zhou, J. Chang, J. Chen, X. Yan, Hf 0.5 Zr 0.5 O 2-based ferroelectric memristor with multi-level storage potential and artificial synaptic plasticity. *Science China Materials* **64**, 727-738 (2020).

4. D. Kim, Y.-R. Jeon, B. Ku, C. Chung, T. H. Kim, S. Yang, U. Won, T. Jeong, C. Choi, Analog synaptic transistor with Al-doped HfO2 ferroelectric thin film. *ACS Applied Materials & Interfaces* **13**, 52743-52753 (2021).

5. L. Chen, T.-Y. Wang, Y.-W. Dai, M.-Y. Cha, H. Zhu, Q.-Q. Sun, S.-J. Ding, P. Zhou, L. Chua, D. W. Zhang, Ultra-low power Hf 0.5 Zr 0.5 O 2 based ferroelectric tunnel junction synapses for hardware neural network applications. *Nanoscale* **10**, 15826-15833 (2018).

6. B. Max, M. Hoffmann, H. Mulaosmanovic, S. Slesazeck, T. Mikolajick, Hafnia-based double-layer ferroelectric tunnel junctions as artificial synapses for neuromorphic computing. *ACS Applied Electronic Materials* **2**, 4023-4033 (2020).

7. V. Mikheev, A. Chouprik, Y. Lebedinskii, S. Zarubin, Y. Matveyev, E. Kondratyuk, M. G. Kozodaev, A. M. Markeev, A. Zenkevich, D. Negrov, Ferroelectric second-order memristor. *ACS applied materials & interfaces* **11**, 32108-32114 (2019).

8. M.-K. Kim, J.-S. Lee, Ferroelectric analog synaptic transistors. *Nano letters* **19**, 2044-2050 (2019).

9. S. Oh, T. Kim, M. Kwak, J. Song, J. Woo, S. Jeon, I. K. Yoo, H. Hwang, HfZrO x-based ferroelectric synapse device with 32 levels of conductance states for neuromorphic applications. *IEEE Electron Device Letters* **38**, 732-735 (2017).

10. M. Ismail, C. Mahata, S. Kim, Forming-free Pt/Al2O3/HfO2/HfAlOx/TiN memristor with controllable multilevel resistive switching and neuromorphic characteristics for artificial synapse. *Journal of Alloys and Compounds* **892**, 162141 (2022).

11. C.-P. Chou, Y.-X. Lin, Y.-K. Huang, C.-Y. Chan, Y.-H. Wu, Junctionless Poly-GeSn ferroelectric thin-film transistors with improved reliability by interface engineering for neuromorphic computing. *ACS applied materials & interfaces* **12**, 1014-1023 (2019).

12. F. Ambriz-Vargas, G. Kolhatkar, M. Broyer, A. Hadj-Youssef, R. Nouar, A. Sarkissian, R. Thomas, C. Gomez-Yáñez, M. A. Gauthier, A. Ruediger, A complementary metal oxide semiconductor process-compatible ferroelectric tunnel junction. *ACS applied materials & interfaces* **9**, 13262-13268 (2017).

13. W. Huang, W. Zhao, Z. Luo, Y. Yin, Y. Lin, C. Hou, B. Tian, C. G. Duan, X. G. Li, A high‐speed and low‐power multistate memory based on multiferroic tunnel junctions. *Advanced Electronic Materials* **4**, 1700560 (2018).

14. H. Sun, Z. Luo, C. Liu, C. Ma, Z. Wang, Y. Yin, X. Li, A flexible BiFeO3-based ferroelectric tunnel junction memristor for neuromorphic computing. *Journal of Materiomics* **8**, 144-149 (2022).

15. M. Pei, J. Qian, S. Jiang, J. Guo, C. Yang, D. Pan, Q. Wang, X. Wang, Y. Shi, Y. Li, pJ-level energy-consuming, low-voltage ferroelectric organic field-effect transistor memories. *The Journal of Physical Chemistry Letters* **10**, 2335-2340 (2019).

16. A. Tsurumaki, H. Yamada, A. Sawa, Impact of Bi deficiencies on ferroelectric resistive switching characteristics observed at p‐type Schottky‐like Pt/Bi1–δFeO3 interfaces. *Advanced Functional Materials* **22**, 1040-1047 (2012).

17. H. Wang, W. Lu, S. Hou, B. Yu, Z. Zhou, Y. Xue, R. Guo, S. Wang, K. Zeng, X. Yan, A 2D-SnSe film with ferroelectricity and its bio-realistic synapse application. *Nanoscale* **12**, 21913-21922 (2020).

18. F. Xue, X. He, Z. Wang, J. R. D. Retamal, Z. Chai, L. Jing, C. Zhang, H. Fang, Y. Chai, T. Jiang, Giant ferroelectric resistance switching controlled by a modulatory terminal for low‐power neuromorphic in‐memory computing. *Advanced Materials* **33**, 2008709 (2021).
